# Proteomic analysis identifies transcriptional cofactors and homeobox transcription factors as TBX18 binding proteins

**DOI:** 10.1371/journal.pone.0200964

**Published:** 2018-08-02

**Authors:** Reginaldo Rivera-Reyes, Marc-Jens Kleppa, Andreas Kispert

**Affiliations:** Institut für Molekularbiologie, Medizinische Hochschule Hannover, Hannover, Germany; Institut de Genetique et Developpement de Rennes, FRANCE

## Abstract

The TBX18 transcription factor is a crucial developmental regulator of several organ systems in mice, and loss of its transcriptional repression activity causes dilative nephropathies in humans. The molecular complexes with which TBX18 regulates transcription are poorly understood prompting us to use an unbiased proteomic approach to search for protein interaction partners. Using overexpressed dual tagged TBX18 as bait, we identified by tandem purification and subsequent LC-MS analysis TBX18 binding proteins in 293 cells. Clustering of functional annotations of the identified proteins revealed a highly significant enrichment of transcriptional cofactors and homeobox transcription factors. Using nuclear recruitment assays as well as GST pull-downs, we validated CBFB, GAR1, IKZF2, NCOA5, SBNO2 and CHD7 binding to the T-box of TBX18 *in vitro*. From these transcriptional cofactors, CBFB, CHD7 and IKZF2 enhanced the transcriptional repression of TBX18, while NCOA5 and SBNO2 dose-dependently relieved it. All tested homeobox transcription factors interacted with the T-box of TBX18 in pull-down assays, with members of the *Pbx* and *Prrx* subfamilies showing coexpression with *Tbx18* in the developing ureter of the mouse. In summary, we identified and characterized new TBX18 binding partners that may influence the transcriptional activity of TBX18 *in vivo*.

## Introduction

T-box (*Tbx*) genes encode a family of proteins that share a highly conserved domain for DNA-binding, the T-box [1, 2]. They act as transcription factors that activate or repress target gene expression upon binding to a conserved short variably spaced and oriented DNA-binding site, the T-box binding element (TBE) [3, 4]. In mammals, genome mining identified 17 members of this gene family that are grouped in five subfamilies depending on sequence conservation. Gene targeting experiments in mice revealed critical functions of some of these genes in the formation and differentiation of the germ layers, and in the development of various organ systems. Mutational analysis in man characterized mutations in *TBX* genes as causes for congenital diseases, demonstrating the importance of the gene family as regulators of developmental programs in mammals (for reviews see [5–8]).

*Tbx18* is a member of a vertebrate specific subgroup within the *Tbx1*-subfamily [9]. Similar to the closely related *Tbx15* and *Tbx22* genes [10–12], *Tbx18* possesses a highly complex and dynamic array of developmental expression sites that in the mouse embryo include the anterior somite halves in the paraxial mesoderm, the developing heart, the mesenchyme surrounding the otic vesicle, the urogenital ridge including the ureteric and prostate mesenchyme, the epicardium, the mandibular/maxillary region, the proximal mesenchyme of the limb buds, and the skin [9, 13–16]. Mice homozygous for an engineered null allele of *Tbx18* died shortly after birth due to severe malformations of the axial skeleton [17]. The capsule and fibrocytes of the inner ear were disrupted, and the sinoatrial node was reduced in size [15, 18]. The epicardium and the coronary vasculature appeared altered [19, 20]. The periductal smooth muscle stromal cells in the prostate were reduced [16]. The ureter was shortened and lacked the smooth muscle coating resulting in hydroureter and hydronephrosis [13]. These defects were traced to independent functions of *Tbx1*8 in patterning and differentiation of the primordia of the affected organs, the somites, the otic mesenchyme, the sinus venosus, and the prostate and ureteric mesenchyme [13–17].

While these studies testified the multitude of developmental functions of the *Tbx18* gene, we still lack insight to what other transcription factors and cofactors TBX18 binds to form transcription regulation complexes, and what target genes are controlled by these complexes at the diverse sites of expression in the embryo. A couple of years ago, our lab started the analysis of the biochemical properties of TBX18 as a transcription factor [21]. We found that the murine protein constitutively localizes to the nucleus and that a classical nuclear localization signal at the N-terminus of the protein accounts for this behavior. PCR based cyclic enrichment of DNA-fragments confirmed that the T-box of TBX18 binds to a combination of conserved TBEs *in vitro*. The T-box does not only mediate DNA-binding, but also constitutes a protein interaction region [21]. Interaction in *in vitro* binding assays was shown with other T-box proteins, including TBX18 itself and TBX15 but also with members of other classes of tissue-specific transcription factors like NKX2.5, GATA4, PAX1, PAX3, PAX9 and SIX1 [21–23].

We further showed that fusion proteins of either TBX18, or its N-terminal, C-terminal and T-box regions with the DNA binding domain of the yeast GAL4 protein repressed transcription of a reporter gene under the control of GAL4 binding sites suggesting that TBX18 acts as a transcriptional repressor and that multiple regions of the protein are able to exert this effect. Removal of a conserved eh1-motif, which acts as a binding site for members of the Groucho (TLE) protein family of transcriptional corepressors abolished the transcriptional repression activity of TBX18 by 50%, stressing the notion that interaction with other cofactors at different sites of the protein confer additional repressive activity to TBX18 [21]. Indications that such interactions exist and are highly relevant at least for the function of TBX18 in the developing ureter *in vivo* were recently provided by the identification of mutations in *TBX18* in patients with congenital anomalies of the kidney and the urinary tract (CAKUT), including ureter-pelvic and vesico-ureteric junction obstruction and hydronephrosis. While one disease-causing mutation led to an exchange of a conserved residue in the T-box, another one led to deletion of the large C-terminal protein domain, and a third resulted in an amino acid exchange in this region. All of these mutant proteins showed reduced transcriptional repression and in the case of the C-terminal deletion mutant even a complete derepression compared to the wildtype protein [24].

Here, we set out to identify and characterize novel protein interaction partners of TBX18 to improve our comprehension of its transcriptional properties. Using an unbiased proteomic screen in 293 cells, we identified transcriptional corepressors and tissue specific transcription factors as binding candidates of TBX18. We validated their binding and present data on their significance for TBX18 transcriptional function.

## Material and methods

### Ethics statement

All animal work conducted for this study was performed according to European and German legislation. Breeding, handling and sacrifice of mice for isolation of embryos was approved by the Niedersächsisches Landesamt für Verbraucherschutz und Lebensmittelsicherheit (Permit Number: AZ33.12-42502-04-13/1356, AZ33.12-42502-04-13/1875).

### Mice

Embryonic day (E) 12.5 embryos for expression analysis were derived form NMRI wild-type mice which were purchased in house from the animal facility of the Medizinische Hochschule Hannover. Three to six mice per cage were housed with *ad libitum* access to food and water under conditions of regulated temperature (22°C) and humidity (50%) and a 12 h light/dark cycle. For timing of the pregnancies, vaginal plugs were checked in the morning after mating and noon was designated as embryonic day (E) 0.5. Female mice were sacrified by cervical dislocation. Embryos and organs were harvested in PBS, decapitated, fixed in 4% paraformaldehyde overnight and stored in 100% methanol at -20°C before further use.

### Semi-quantitative reverse transcription PCR

Cells were grown on 10 cm dishes until they reached confluence. Total RNA was extracted with peqGOLD RNApure (PeqLab), and first-strand cDNA synthesis was performed with RevertAid reverse transcriptase (Fermentas). For quantitative PCR amplification of *Tbx18*, the forward primer 5’-GGTGGCAGGTAATGCTGACT and the reverse primer 5’-ACTTGCATTGCCTTGCTTGG were annealed at 56°C for 30 min, before elongation occurred at 72°C for 30 sec. The number of cycles was adjusted to the mid-logarithmic phase. For normalization, *Gapdh* was used (forward primer: 5’- ACCACAGTCCATGCCATCAC, reverse primer: 5’- TCCACCACCCTGTTGCTGTA, annealing at 56°C for 30 sec, elongation time 20 sec [25]. Quantification was performed with ImageJ 1.47 [26]. Assays were repeated at least three times in duplicate, and statistical analysis was done as previously described [27].

### Identification of TBX18 interacting proteins in 293 cells

A cDNA fragment encoding a triple FLAG tag was generated by assembly PCR from several overlapping oligonucleotides. *Nco*I and *Sma*I sites were introduced at the ends to replace the single FLAG tag in *p*.*EF1α*.*FLAG*.*BioTag* (kind gift of A.P. McMahon) [28] with this fragment. A cDNA fragment with the open reading frame of *Tbx18* was amplified from a full-length *Tbx18* cDNA [9], and cloned into the *BamH*I/*Xba*I site of the resulting *p*.*EF1α*.*3xFLAG*.*BioTag* plasmid generating the *p*.*EF1α*.*3xFLAG*.*BioTag*.*Tbx18* plasmid. A *IRES*.*NLS*.*EGFP*.*BirA*.*BGHpA* fragment was released from *p*.*EF1α*.*Six2DE*.*IRES*.*NLS*.*EGFP*.*BirA*.*BGHpA* (kind gift of A.P. McMahon) [28] and cloned into the *Nhe*I site of *p*.*EF1α*.*3xFLAG*.*BioTag*.*Tbx18* yielding the plasmid *p*.*EF1α*.*3xFLAG*.*BioTag*.*Tbx18*.*IRES*.*NLS*.*EGFP*.*BirA*.*BGHpA*. This plasmid encodes a TBX18 protein fused to a N-terminal triple FLAG tag and a biotinylation signal peptide while also expressing the bacterial BirA enzyme required for biotinylation. 15 μg of the *p*.*EF1α*.*3xFLAG*.*BioTag*.*Tbx18*.*IRES*.*NLS*.*EGFP*.*BirA*.*BGHpA* and of the mock plasmid *p*.*EF1α*.*IRES*.*NLS*.*EGFP*.*BirA*.*BGHpA*, respectively, were transfected into 293 cells grown in a 15-cm dish at 70% confluence at the day of transfection using the calcium phosphate method [29]. Transfection efficiency was verified by fluorescence microscopy to be 80%. The next day the cells were expanded on 10 x 15-cm cell culture dishes and cultured for two more days until they reached 90–95% confluence. Nuclear extracts were prepared from these cells and 10 mg protein content subjected to tandem affinity purification using first anti-FLAG M2 antibody (Sigma, #F3165) coupled to Protein A agarose beads (Santa Cruz, #sc-2001), with subsequent elution with a triple FLAG peptide (Sigma, #F4799), and second, a Strep-Tactin Superflow (IBA, #2-1206-002) binding column as previously published [30]. The purified complexes were resolved by SDS-PAGE, protein bands were excised and sent to the MHH Proteomics Facility where they were analyzed by liquid chromatography-mass spectrometry (LC-MS).

### Sample preparation for MS analysis

Proteins were mixed, alkylated by acrylamide and further processed as described [31]. Peptide samples were analysed with a shot-gun approach and data dependent analysis in a LC-MS system (RSLC, LTQ Orbitrap Velos, both Thermo Fisher) as recently described [31]. Raw MS data were processed using Proteom discoverer 1.4 (Thermos Scientific) and Max Quant software (version 1.5) [32] and a data base containing human and viral proteins and common contaminants. Proteins were stated identified by a false discovery rate of 0.01 on protein and peptide level.

### Bioinformatic analysis of MS datasets

Gene Ontology (GO) analysis was performed on the 143 proteins identified from TBX18 immunoprecipitation experiments to determine the enrichment of annotated molecular functions (MF), biological processes (BP) and cellular components (CC) using the Database for Annotation, Visualization and Integrated Discovery (DAVID) web program [33].

### Expression constructs

Vectors for expression of proteins in 293 cells, for *in vitro* translation of proteins and for transactivation assays were acquired from researchers, companies or generated in house as detailed in S1 Table. Cloning was done by PCR amplification of ORFs from donor plasmids and insertion of the restricted fragments into suitable sites in plasmids for *in vitro* expression (*pSP64*.*G*.*Myc/HA*) [21], and further shuttling into the eukaryotic expression plasmid *pcDNA3* (Invitrogen).

### *In vitro* translation of radioactively labeled proteins

We used a coupled *in vitro* transcription/translation system from reticulocyte lysates (TNT SP6 or T7 Quick Coupled Transcription/Translation System, Promega) to generate proteins *in vitro*. 2 μg of the plasmid with a SP6 or T7 promoter were used for 50 μl reaction set-ups. ^35^S labeled Methionine/Cysteine (Hartmann Analytic, Braunschweig, Germany) was added to a final concentration of 0.2–0.4 μCi/μl and reactions were incubated for 1.5 hr or 3 hr for longer proteins at 30°C.

### GST-TBX18 fusion protein production

GST-TBX18 fusion proteins were produced as previously shown [21]. Briefly, the fragments encoding the N-terminal (aa 1–157), N-terminal plus T-box (aa 1–345), T-box (aa 148–345) and C-terminal region (aa 336–613) of TBX18 were inserted into the *pGEX-4T3* plasmid and transformed into *E*.*coli* BL-21 bacteria. After culture and IPTG induction, the bacteria were lysed and proteins were prepared by batch-purification using Glutathion Sepharose 4B beads (GE Healthcare, Chicago, Illinois). The protein content of the recovered beads was resolved by SDS-PAGE and visualized by Coomassie staining and the volumes of beads to achieve a standardized content of all of the GST-TBX18 fusion proteins were calculated with the help of ImageJ [26].

### GST pull-down assays

The GST pull-down assays were performed as previously reported [21]. One or more 10-cm culture plates of 293 cells were transfected with 20 μg of eukaryotic expression plasmid using the calcium phosphate method. 2 days after transfection, proteins were extracted from each plate with 250 μl of NP-40 buffer with Phostop and cOmplete inhibitors (Roche) and a 1/10 aliquot was checked for protein expression by Western Blot. The rest of the protein extract was incubated with the standardized bead volumes of the GST-TBX18 domain fusion proteins for two hours before washing 3 times with pull-down buffer (20 mM HEPES, pH 7.9, 100 mM NaCl, 10 mM KCl, 5 mM MgCl_2_, 0.5 mM EDTA, 5% glycerol, 0.05% Triton X-100, 1 mM dithiothreitol). The beads were boiled in 1x Laemmli buffer and the proteins were separated by SDS-PAGE. After blotting onto PVDF membranes, interactions were detected by Western Blot with anti-HA, anti-MYC and anti-FLAG antibodies (#ab1265 and #ab62928, Abcam, #F3165, Sigma).

For the GST pull-down with *in vitro* translated proteins, 20 to 40 μl of transcription/translation reaction were incubated with the standardized bead volume of each of the GST-TBX18 domain fusion proteins for two hours before washing 3 times with pull-down buffer. Analysis of bound protein was performed by SDS-PAGE followed by drying of the gels onto cellulose filters. Autoradiographic detection was performed by exposing the filters for 24 hr to Fujifilm BAS-IP MS 2025 phosphorescent imaging plates and scanning them in a Fujifilm FLA7000 Laser scanner.

### Cell culture and transient transfections

293 cells were purchased (ACC 305, DSMZ, Braunschweig, Germany), all other cell lines were kindly provided by T. von Hahn (Medizinische Hochschule Hannover, Germany). Cell cultures were handled under strict sterile conditions. 293 cells were cultured in standard DMEM medium (GIBCO) containing 10% fetal bovine serum (Biochrom) and kept in an incubator at 37°C with 5% CO_2_. The transient transfections were performed with the calcium phosphate method as previously described [29]. The *pd2E*.*GFP-N1* plasmid was independently transfected to check for efficiency of transfection, which was verified by epifluorescence microscopy.

### Immunofluorescence analysis

293 cells were cultured in 12 well plates and transfected with *pcDNA3*.*1*.*TBX18ΔNLS* (500 ng), *pcDNA3*.*1*.*TBX18* (500 ng) or *pcDNA3*.*1* expression plasmids of the candidate proteins (500 ng). For recruitment assays, co-transfections were performed with either *pcDNA3*.*1*.*TBX18ΔNLS* (250 ng) or *pcDNA3*.*1*.*TBX18* (250 ng) plasmids, *and pcDNA3*.*1* expression plasmids of the candidate proteins (250 ng). After 2 days, immunofluorescent detection of TBX18 and the candidate proteins using the corresponding tags as previously reported [21] and imaged with a DM6000 microscope (Leica).

### Reporter constructs and transactivation assays

To generate a reporter for TBX18-dependent transcriptional activity, we annealed the single-stranded DNA fragments 5´-GATCCGGTGCAGTAGGTGTGAAATCGCACCTGGGGA-3´ and 5´-GATCTCCCCAGGTGCGATTTCACACCTACTGCACCG-3´ and ligated the resulting double-stranded fragment into the *Bgl*II site of the *pGL3*.*Promoter* vector (Promega) to obtain *pGL3*.*Prom*.*Tbx18BS2*. Dual luciferase assays were performed in 293 cells in duplicates. Cells were seeded to 70% confluence in 6-well plates and 1 day later transfected using the calcium phosphate method with 2.5 μg of plasmid mix. 100 ng of *pRL-TK* were used for normalization, 250 ng of *pGL3*.*Prom*.*Tbx18BS2* were used to measure transcriptional activity, 250 ng of *pcDNA3*.*Tbx18* plasmid were used to evaluate the effect of TBX18, and in separate wells, additional 25, 250 and 500 ng of vectors encoding the interaction candidates were added to analyze the effect on TBX18 regulation of transcription. *pcDNA3* plasmid was used to fill up to 2.5 μg. After 12 h, medium was refreshed and 1 day later the cells were extracted and analyzed according to the Dual-Luciferase Reporter Assay System kit (Promega #E1910) in two technical replicates.

### RNA *in situ* hybridization

10-μm paraffin sections of the posterior trunk region of embryonic day (E) 12.5 wildtype NMR mice containing the proximal ureter region were subjected to RNA hybridization with digoxigenin-labeled antisense riboprobes as previously described [34].

## Results

### Mass spectrometry analysis identifies novel TBX18 interacting proteins in 293 cells

**Fig 1:**
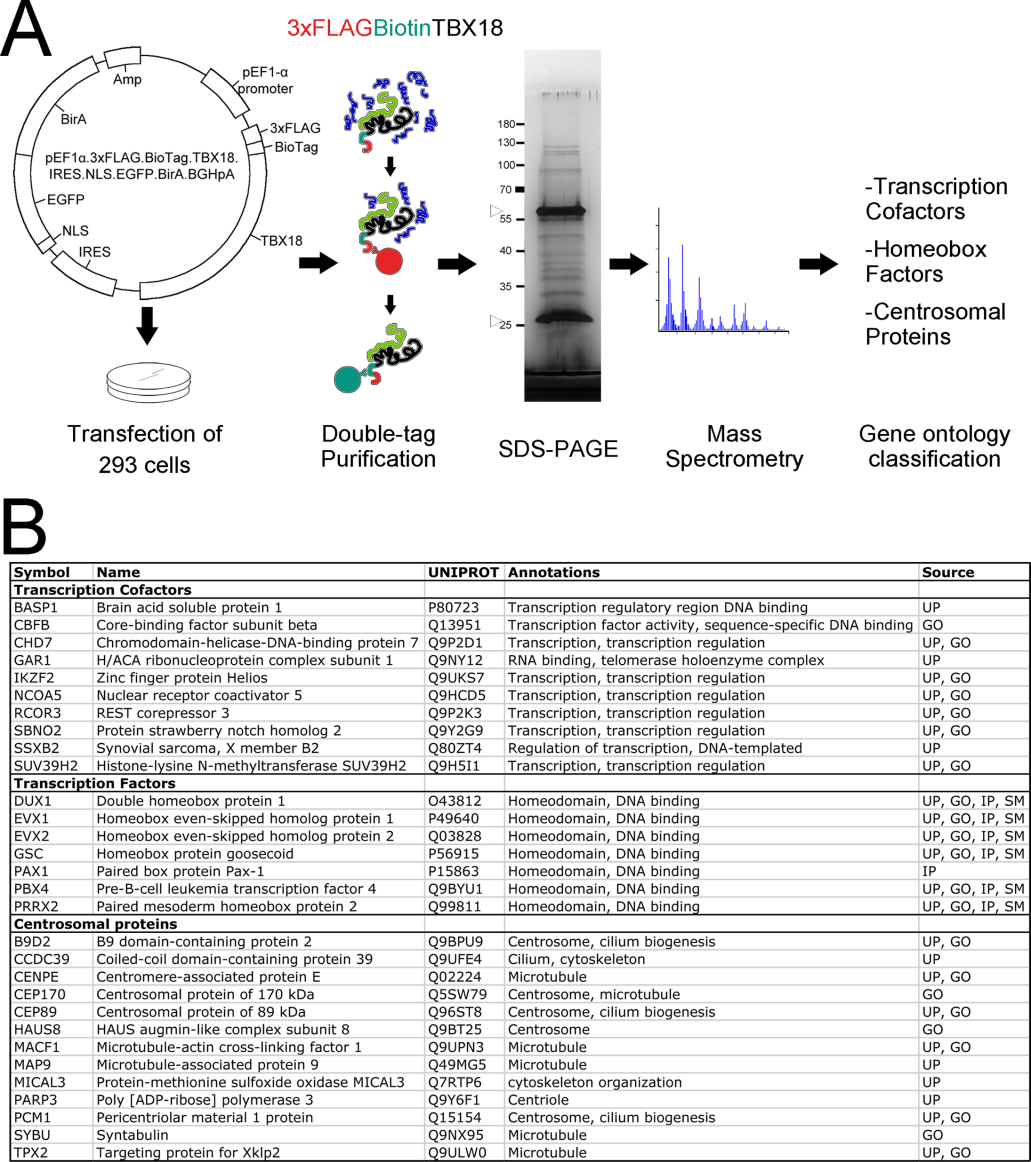
Mass spectrometry analysis identifies novel TBX18 interacting proteins in 293 cells. (A) Diagram of the identification strategy. A construct encoding mouse TBX18 protein fused to a N-terminal triple FLAG tag followed by a biotinylation signal peptide while also encoding the bacterial BirA enzyme required for biotinylation was transfected into 293 cells. Protein complexes containing TBX18 were purified from nuclear cell extracts by a two-step affinity purification strategy using Anti-FLAG (red) and anti-Biotin chromatography (green). The purified protein complexes were resolved by SDS-PAGE. Silver staining of a sample aliquot is displayed, arrowheads show IgG heavy and light chains. After extraction, they were subjected to LC-MS analysis. Proteins were functionally classified and clustered. (B) Gene annotation enrichment analysis uncovered clusters of transcriptional cofactors, transcription factors of the homeobox family, and centrosomal proteins. Gene ontology analysis is provided. UNIPROT: Uniprot accession number. Database annotations are UP: Uniprot, GO: Gene Ontology, IP: Interpro, SM: Smart.

Expression of *Tbx18* occurs at low levels in small progenitor pools of mammalian organ primordia hampering biochemical approaches to isolate endogenous complexes of TBX18 and interacting proteins. Since transcription factors exert transcriptional modulation functions upon expression in most cultured cells, we deemed that cell lines represent a more easily accessible source for the identification of at least some of the proteins that bind to TBX18 and affect its transcriptional activity. We chose 293 cells as a source for our proteomic screen since these cells are easy to grow and transfect, and are derived from an embryonic organ, the human kidney. Moreover, these cells express *TBX18* arguing that binding partners of TBX18 naturally occur in these cells (S1 Fig). Since available antibodies proved unsuitable for precipitation of endogenous protein complexes of TBX18 in our hands, we decided to transiently overexpress a version of TBX18 tailored for tandem affinity precipitation of native complexes [30]. For this, we transfected an expression construct encoding the mouse TBX18 protein fused to a N-terminal triple FLAG tag followed by a biotinylation signal peptide and the bacterial BirA enzyme required for biotinylation (Fig 1A). After cell expansion and nuclear extraction, the protein complexes containing TBX18 were purified by a two-step affinity purification strategy using Anti-FLAG antibody coupled to Protein A agarose beads and subsequent streptavidin affinity chromatography [28, 30].

The purified protein complexes were recovered by heat treatment and resolved by SDS-PAGE. The polyacrylamide gels of three independent experiments and controls were sent to the Hannover Medical School Proteomics Facility for protein extraction and subsequent LC-MS analysis (Fig 1A). Fragments of 143 human proteins were identified in any of the three independent IP-MS analyses we did but not in the control which lacked exogenous TBX18 expression (S2 Table). Ontological classification of annotated functions, biological processes and functional domains revealed a list of 84 enriched terms, in which nucleosome, homeobox, DNA-binding, nuclear chromatin were amongst the most highly significant ones (S3 Table). Furthermore, DAVID functional clustering with high stringency settings grouped these terms into 5 clusters, with nucleosome/chromatin and homeobox/DNA-binding/transcription related proteins having the highest enrichment scores (S4 Table). Histone H2A was by far the most strongly enriched protein indicating together that overexpressed TBX18 associates with other transcriptional regulatory proteins on chromatin in 293 cells. We manually mined the available literature for each candidate and curated a list of 10 proteins (BASP1, CBFB, CHD7, GAR1, IKZF2, NCOA5, RCOR3, SBNO2, SSXB2 and SUV39H2) with a functional assignment as transcriptional cofactors and a second list of 13 proteins (DUX1, DUX3, DUX4, DUX4L2, DUX4L4, DUX4L9, DUX5, EVX1, EVX2, GSC, PAX1, PRRX2, PBX4) characterized as tissue-specific transcription factors of the homeobox superfamily. Functional clustering and individual literature cross-referencing unexpectedly identified 13 proteins (B9D2, CCDC39, CENPE, CEP170, CEP89, HAUS8, MACF1, MAP9/ASAP, MICAL3, PARP3, PCM1, SYBU, TPX2) with a centrosomal and microtubule association (Fig 1B). Based on the recent report that the transcription factor ATF5 has an independent centrosomal function [35], we wished to determine whether these proteins might relate to an additional unexpected cellular function of TBX18. We rejected intermediate filament, keratin, actin filament, iron-sulfur, translation elongation, kinase, cell adhesion and ankyrin containing proteins for further validation as the established cytosolic or extracellular localization of these proteins point to an unspecific nature of the interaction.

### Transcriptional cofactors bind to TBX18 in the nucleus of 293 cells

**Fig 2:**
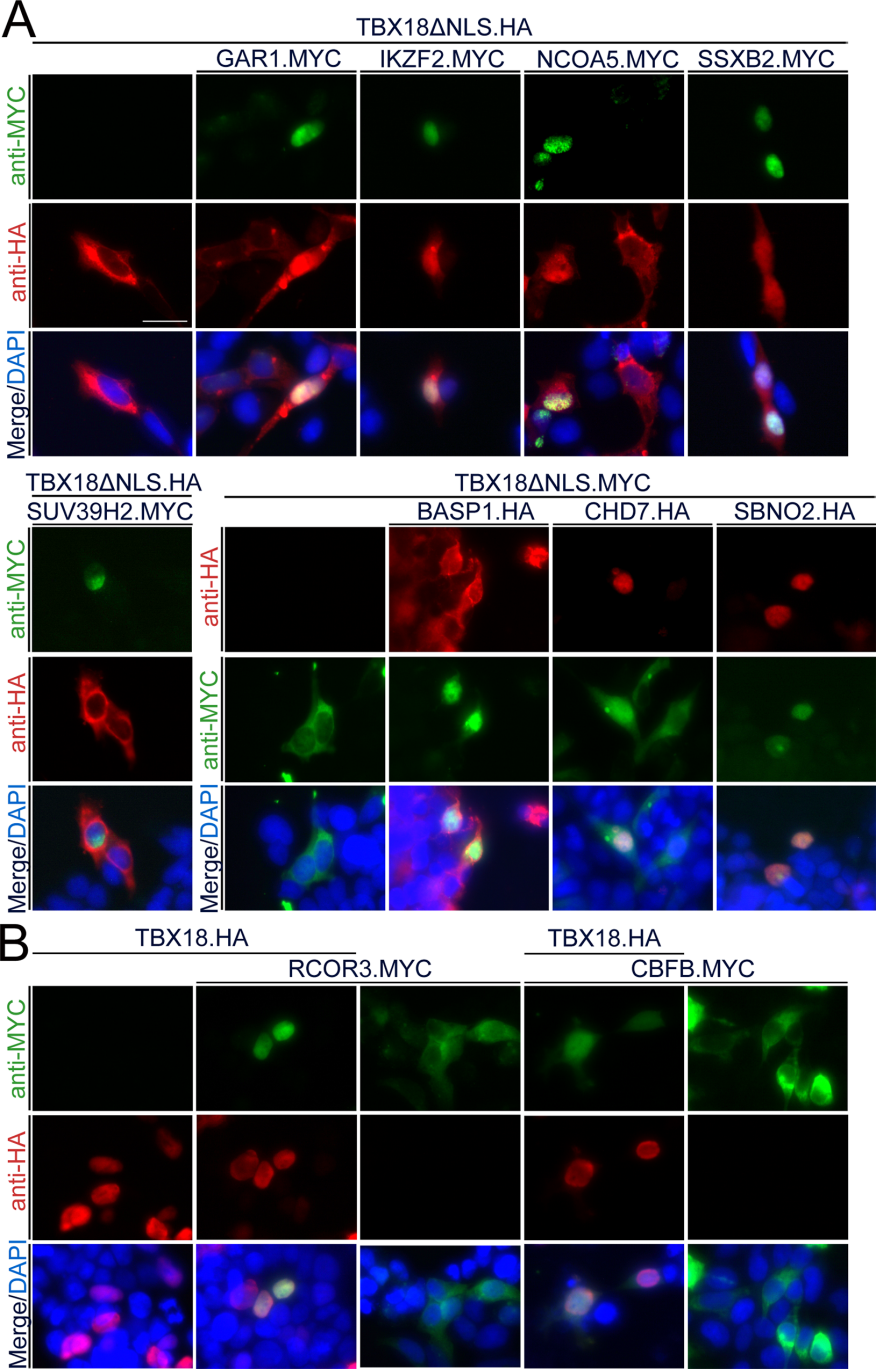
TBX18 colocalizes with most of the candidate transcriptional cofactors in the nucleus of 293 cells. (A) 293 cells were transfected with expression constructs for MYC-tagged GAR1, IKZF2, NCOA5, SSXB2 or SUV39H2 (*green)* in the presence of HA-tagged TBX18 lacking the nuclear localization signal (TBX18ΔNLS, *red*), or with expression constructs for HA-tagged BASP1, CHD7 or SBNO2 (red) in the presence of MYC-tagged TBX18ΔNLS (green). Immunofluorescence analysis shows that NLS-deficient TBX18 protein is efficiently shuttled from the cytoplasm to the nucleus by all candidate proteins except SUV39H2. (B) 293 cells were transfected with expression constructs for MYC-tagged RCOR3 or CBFB (*green)* in the presence of HA-tagged full-length TBX18 protein (TBX18ΔNLS, *red*). Immunofluorescence analysis shows that TBX18 protein recruits RCOR3 and CBFB from the cytoplasm into the nucleus. DAPI, 4,6-diamidino-2-phenylindole nuclear counterstain. Scale bar length is 25 μm.

We validated the interactions of TBX18 with the candidate transcriptional cofactors using a number of independent assays. We started with interrogating whether in fact TBX18 colocalizes with and binds to these proteins in 293 cells. For this, we first transfected expression constructs for full length MYC- or HA-tagged candidate proteins, confirmed protein integrity by Western blot and characterized their localization by anti-MYC or anti-HA immunofluorescence analysis (S5A Table, S2A Fig). In addition to TBX18, BASP1, CHD7, GAR1, IKZF2, NCOA5, SBNO2, SSXB2 and SUV39H2 localized to the nucleus, while CBFB and RCOR3 were found in the cytoplasm (S2B Fig). To test, whether TBX18 productively interacts with the candidate cofactors in 293 cells, we made use of a nuclear recruitment assay that we previously developed [21]. It is based on the identification of a classical nuclear localization signal (NLS) at the N-terminus of the TBX18 protein. When this NLS is deleted, the resulting protein (TBX18ΔNLS) is excluded from the nucleus but can be shuttled back to this compartment by binding to a protein which carries such a signal. Upon coexpression of TBX18ΔNLS and those candidate cofactors that showed nuclear localization in 293 cells, we observed a nuclear translocation of TBX18ΔNLS in the presence of BASP1, CHD7, GAR1, IKZF2, NCOA5, SBNO2 and SSXB2 proteins while coexpression of SUV39H2 left the cytoplasmic localization of TBX18ΔNLS unchanged (Fig 2A). To study TBX18 interaction with CBFB and RCOR3 that reside in the cytoplasm, we modified this assay by cotransfecting expression constructs for full-length TBX18, and CBFB or RCOR3, respectively. We observed that coexpression of TBX18 sufficed to shuttle the two proteins into the nucleus (Fig 2B). Together, this shows that TBX18 interacts with all transcriptional cofactor candidates except SUV39H2 in 293 cells.

### Several transcriptional cofactors bind to TBX18 in a physically direct manner

**Fig 3:**
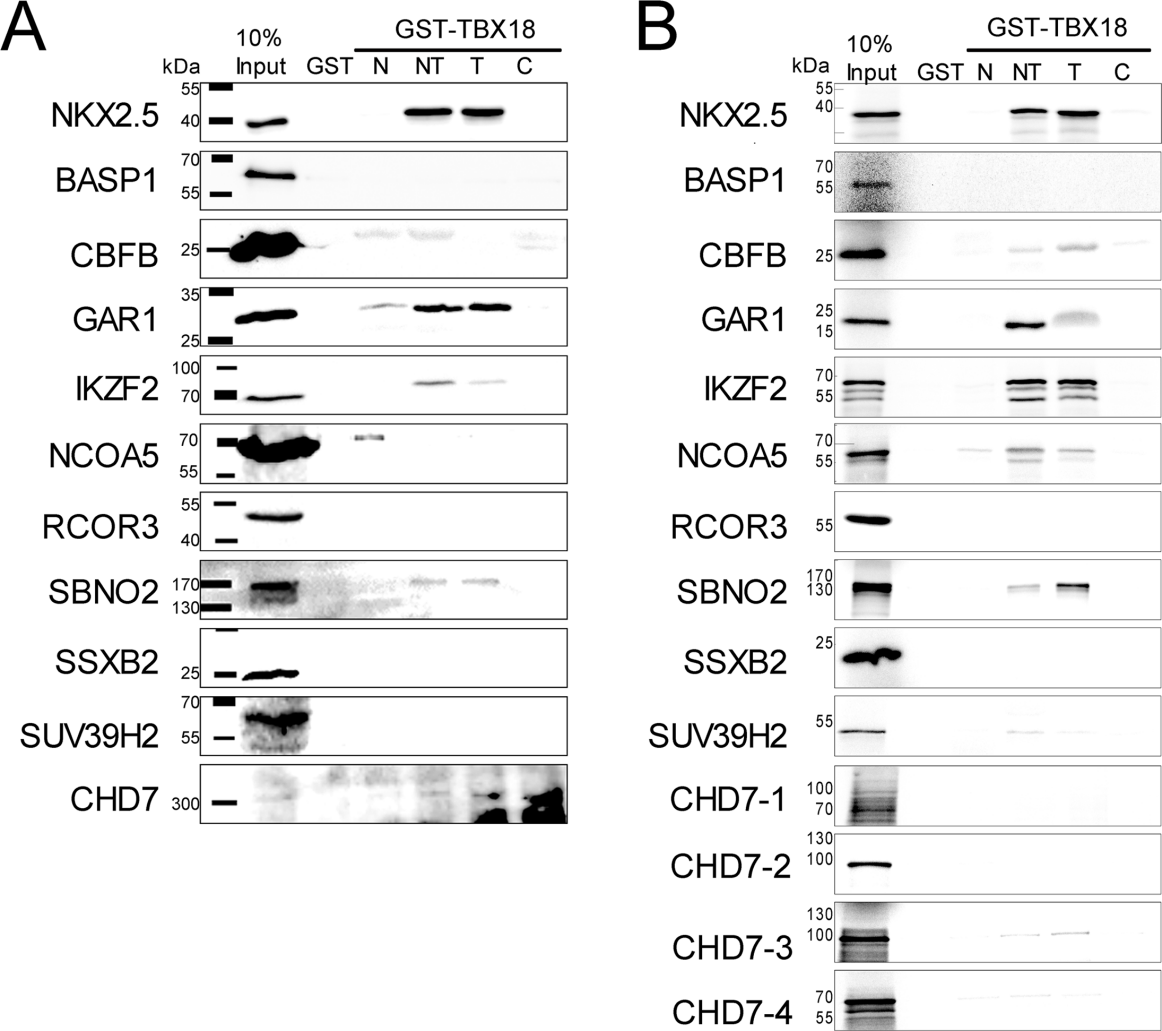
TBX18 physically interacts with candidate transcriptional cofactors preferentially via its T-box. (A) Western blot analysis of pull-down assays performed with GST and fusion proteins of GST with N-, N+T (NT), T-, and C-domains of TBX18 obtained from *E*. *coli* extracts, and protein extracts from 293 cells transfected with MYC- or HA-tagged full-length expression constructs of candidate transcriptional cofactors. Detection was performed with anti-MYC immunohistochemistry for CBFB, GAR1, IKZF2, NCOA5, RCOR3, SSXB2 and SUV39H2, and anti-HA immunohistochemistry for NKX2.5, BASP1, SBNO2 and CHD7. (B) Autoradiographic analysis of pull-down assays performed with the same GST-TBX18 fusion proteins and reticulocyte lysates programmed for *in vitro* translation of ^35^S-labelled full-length candidate transcriptional cofactors. CBFB, GAR1, IKZF2, SBNO2, NCOA5 and CHD7 interact with the T-box of TBX18 as does the control protein NKX2.5. Due to the large size of CHD7, subfragments were expressed by *in vitro* translation and used in the pull-down assay. CHD7-1, amino acid residues 1–799; CHD7-2, 732–1567; CHD7-3, 1533–2380; CHD7-4, 2325–2997.

To independently assess the physical interaction of TBX18 with the candidate transcriptional cofactors, we performed pull-down assays using bacterially expressed GST-TBX18 proteins that were incubated with lysates from 293 cells transfected with expression constructs for MYC- or HA tagged candidate proteins (S5A Table). Since GST-TBX18 full-length protein could not be expressed in bacteria, we used a previously described series of bacterially expressed fusion proteins of GST with the N- and C-terminal region and the T-box of TBX18 (S3 Fig) which simultaneously allowed to delimit the interaction domain with TBX18 [21]. Under the chosen experimental conditions, NKX2.5 bound well to the T-box of TBX18 as previously reported [21]. From the candidate cofactors, BASP1, RCOR3, SSXB2 and SUV39H2 did not bind to TBX18. CBFB bound to the N-, and C-terminal regions and the T-box, GAR1 to the T-box and weakly to the N-terminal region, IKZF2 and SBNO2 bound to the T-box, NCOA5 to the N-terminal region and more weakly to the T-box, the large CHD7 protein bound to the N- and T-box regions of TBX18 (Fig 3A).

Since 293 cell lysates represent a highly complex protein mix, it is conceivable that binding of the cofactors to TBX18 is mediated by additional proteins from the lysates. Furthermore, proteins from the lysate may preferentially bind to TBX18 thereby masking binding sites for our candidate cofactors. To address these concerns, we used reticulocyte lysates as an alternative and less complex source for the synthesis of the candidate cofactors (S5B Table). We labeled the proteins by addition of ^35^S-Methionin to the *in vitro* translation reaction, and visualized their interaction with GST-TBX18 fusion proteins by autoradiographic imaging after SDS-PAGE of pulled-down complexes. Again, we did not find binding of BASP1, RCOR3, SSXB2 and SUV39H2 to TBX18 protein fragments. CBFB bound to the T-box and weakly to the C-terminal region, GAR1, IKZF2, and SBNO2 bound to the T-box of TBX18, NCOA5 and a subfragment of CHD7 ranging from 1533–2380 [36] bound to the T-box and weakly to the N-terminal region (Fig 3B). Together, these pull-down assays identify the T-box as the TBX18 subregion with which GAR1, IKZF2, SBNO2, NCOA5 and CHD7 strongly and most likely directly interact.

### Some of transcriptional cofactors influence TBX18 transcriptional activity i*n vitro*

**Fig 4:**
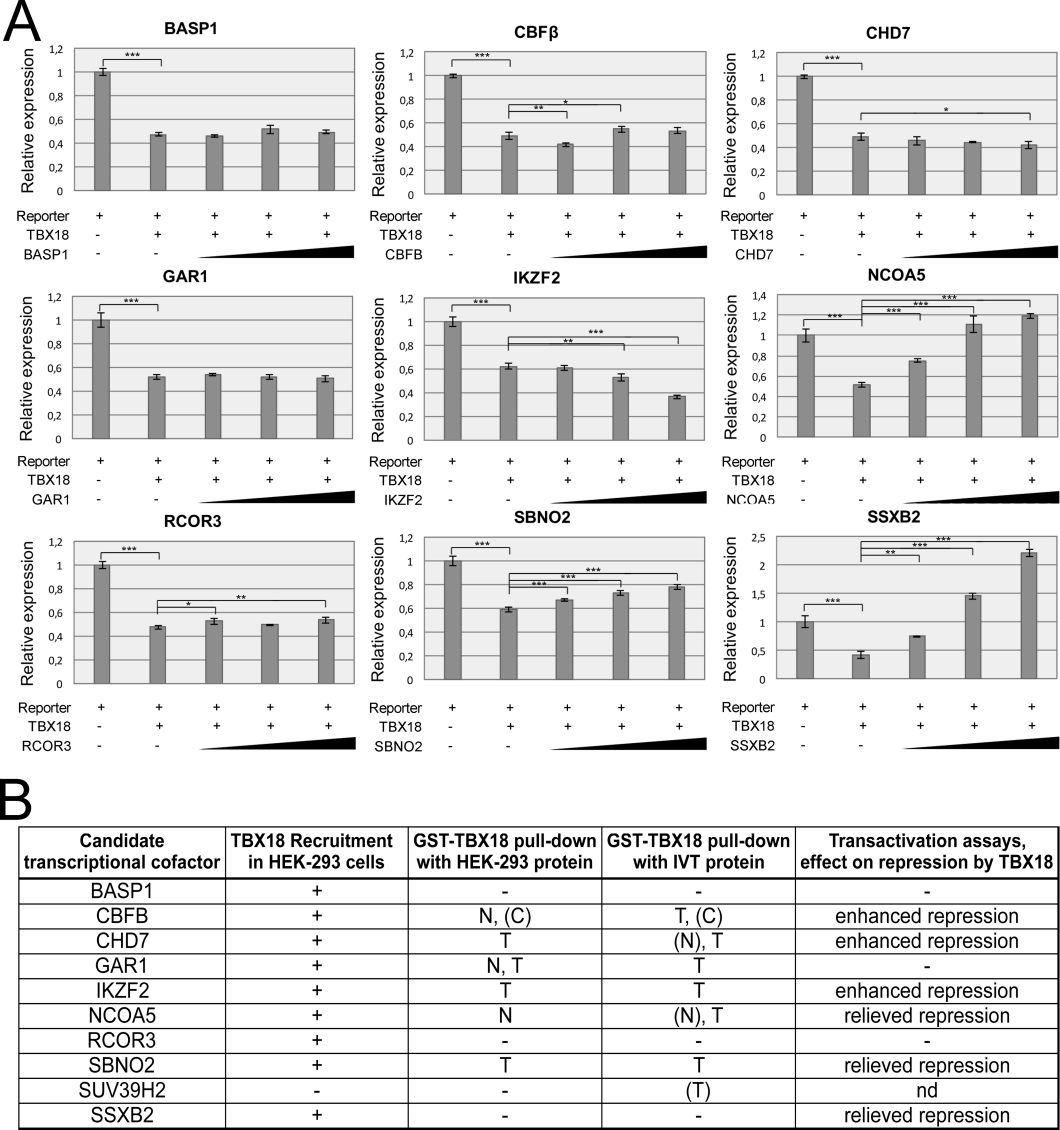
Candidate transcriptional cofactors modify the transcriptional repression activity of TBX18. (A) Luciferase assays from extracts of 293 cells cotransfected with 250 ng of the *pGL3*.*Prom*.*Tbx18BS2* reporter plasmid, 250 ng of the TBX18 expression plasmid *pcDNA3*.*Tbx18* and 25, 250 or 500 ng of plasmids for expression of the transcriptional cofactors. Luciferase activity is normalized to a cotransfection of the reporter plasmid with an empty *pcDNA3* expression vector. TBX18 represses luciferase activity to about 50% of the control value. CBFB, CHD7, IKZF2, RCOR3 further augment repression by TBX18; NCOA5, SBNO2 and SSXB2 relieve TBX18 repression activity. Values are displayed as mean ± sd. * P≤0.05 ** P≤0.01 ***P≤0.001; two-tailed Student's t-test. (For statistical values see S6 Table). (B) Summary of TBX18 binding and activation assays for transcriptional cofactor candidates. Three cofactors (CBFB, CHD7, IKZF2) reliably interact with the T-box of TBX18 and repress transcription. Two cofactors interact with the TBX18 T-box but relieve repression.

We next performed luciferase reporter assays in 293 cells to analyze whether and how the candidate cofactors modulate TBX18 transcriptional activity. Cotransfection of an expression plasmid of TBX18 and a reporter plasmid in which we cloned a palindromic repeat of two TBEs which we previously described [21] in front of an SV40 minimal promoter and a firefly luciferase gene, resulted in 50% repression of luciferase activity compared to an empty expression vector. Cotransfection of increasing amounts of expression plasmids for BASP1 and GAR1 did not affect repression by TBX18. CBFB and CHD7 led to a weakly enhanced repression at least at one of the chosen concentrations. IKZF2 addition resulted in a dose-dependent increase of repression. In contrast, RCOR3 slightly relieved repression, while NCOA5, SBNO2 and SSXB2 dose-dependently counteracted the repressive activity of TBX18 (Fig 4A, S6 Table). Together, these assays identify CBFB, CHD7 and IKZF2 as transcriptional cofactors that bind the T-box of TBX18 directly and enhance (at least moderately) transcriptional repression of TBX18, while NCOA5 and SBNO2 bind but relieve repression (Fig 4B).

### Homeobox transcription factors bind to the T-box of TBX18 and are coexpressed during ureter development

**Fig 5:**
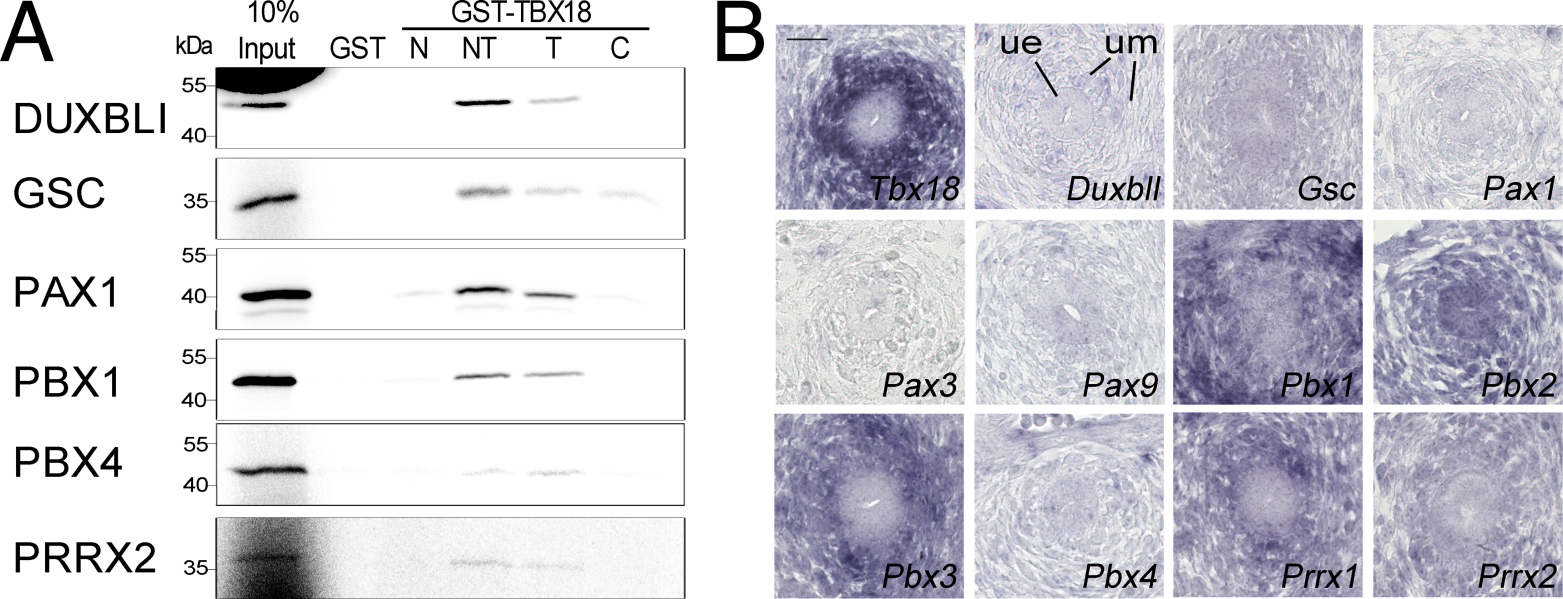
Homeobox transcription factors directly bind to the T-box of TBX18 and are coexpressed with *Tbx18* during ureter development. (A) Autoradiographic analysis of pull-down assays of GST and of fusion proteins of GST with N-, N+T (NT)-, T-, and C-domains of TBX18 from *E*. *coli* extracts, and reticulocyte lysates programmed for *in vitro* translation of ^35^S-labelled full-length candidate transcriptional cofactors. DUXBL1, GSC, PAX1, PBX1, PBX4 and PRRX2 interact with the T-box of TBX18. (B) Comparative RNA *in situ* hybridization analysis on transverse sections of the proximal ureter of E12.5 embryos of *Tbx18* and of genes encoding homeobox transcription factor candidates and related subfamily members. ue, ureteric epithelium, um, ureteric mesenchyme. Scale bar length is 25 μm.

Our proteomic screen identified 13 tissue-specific transcription factors that all featured a homeobox as DNA-binding region (Fig 1B). To validate the binding of these proteins to TBX18, we used the pull-down assay with GST-TBX18 fusion proteins and reticulocyte lysates programmed for the translation of radioactively labeled full-length proteins (S5C Table). Strikingly, all tested homeobox transcription factors (DUXBL1, the unique homologous mouse gene for the human DUX family, GSC, PAX1, PBX1, PBX4, PRRX2) bound to the T-box of TBX18. GSC additionally weakly interacted with the C-terminal region (Fig 5A). To determine a possible *in vivo* relevance of this finding, we analyzed whether the encoding genes as well as related members of the specific subfamily are coexpressed with *Tbx18* in the developing ureter. *In situ* hybridization on sections of E12.5 mouse ureters showed that *Pbx1*, *Pbx2*, *Pbx3*, *Prrx1* and *Prrx2* are coexpressed with *Tbx18* in the mesenchymal compartment of the organ, holding the promise of a functional interaction in ureter development (Fig 5B).

### TBX18 does not bind candidate proteins at the centrosome

**Fig 6:**
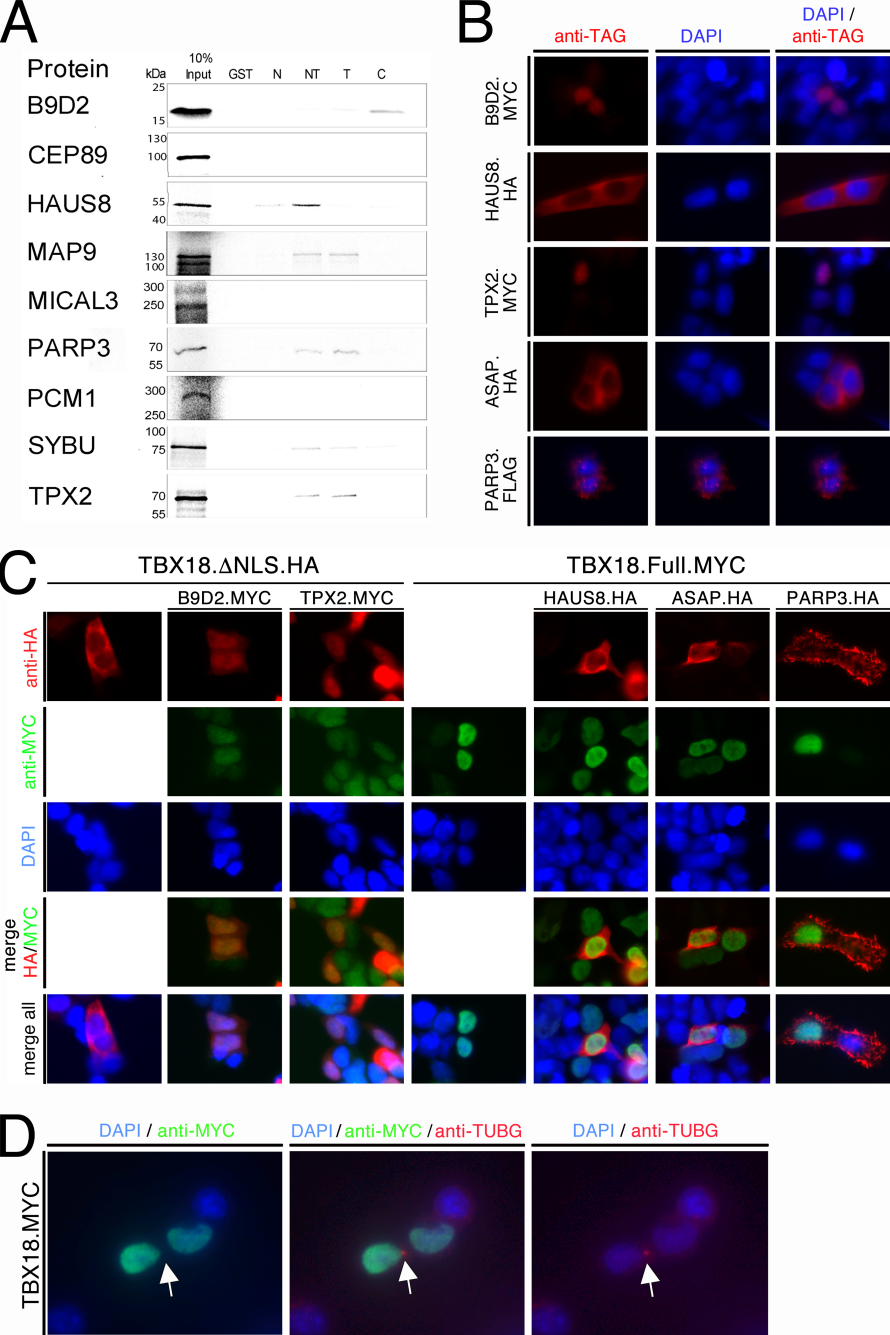
TBX18 does not bind to candidate interaction partners at the centrosome. (A) Autoradiographic analysis of pull-down assays of GST and fusion proteins of GST with N-, N+T- (NT), T-, and C-domains of TBX18 from *E*. *coli* extracts, and reticulocyte lysates programmed for *in vitro* translation of ^35^S-labelled full-length candidate centrosomal proteins. B9D2, HAUS8, MAP9, PARP3, SYBU and TPX2 interact with the T-box of TBX18; B9D2 and SYBU additionally bind to the C-, HAUS8 to the N-terminal domain. (B) Immunofluorescent staining against epitope tags of candidate centrosomal proteins expressed in 293 cells reveals nuclear localization of B9D2 and TPX2, cytoplasmic localization of HAUS8 and ASAP, and confinement of PARP3 to cellular protrusions. (C) 293 cells were transfected with expression constructs for MYC-tagged B9D2 and TPX2 (*green)* in the presence of HA-tagged TBX18 lacking the nuclear localization signal (TBX18ΔNLS, *red*), or with expression constructs for HA-tagged HAUS8, ASAP and PARP3 (*red*) in the presence of MYC-tagged full-length TBX18 (*green*). Immunofluorescence analysis shows that NLS-deficient TBX18 protein is efficiently shuttled from the cytoplasm to the nucleus by B9D2 and TPX2 while the extranuclear localization of HAUS8, ASAP and PARP3 is unaffected by coexpression of full-length TBX18. (D) 293 cells were transfected with an expression construct for MYC-tagged TBX18 (*green)*. Co-immunofluorescence analysis shows that TBX18 protein does not localize to the centrosome marked by TUBG expression (*red*) but to the nucleus. Scale bar length is 25 μm.

To evaluate the possibility of direct interactions between TBX18 and the identified centrosomal proteins (Fig 1B), we performed GST pull-down assays again with *in vitro* translated proteins as described (S5D Table). Interaction was found between TBX18´s T-box and MAP9, PARP3, SYBU and TPX2. HAUS8 bound to the N-terminal domain, while B9D2 and (weakly) SYBU additionally bound to the C-terminal domain (Fig 6A). We next checked whether in fact TBX18 colocalizes with and binds to some of these proteins in 293 cells. For this, we first transfected expression constructs for full-length epitope-tagged candidate proteins, confirmed protein integrity by Western blot and characterized their localization by immunofluorescence analysis against their tags (S5E Table, S4 Fig). To our surprise, we did not detect any centrosomal accumulations but found that B9D2 and TPX2 localized to the nucleus, HAUS8 and ASAP to the cytoplasm, and PARP3 to cellular protrusions (Fig 6B). Upon coexpression of TBX18ΔNLS with B9D2 and TPX2, we observed a nuclear translocation of TBX18ΔNLS. In contrast, full-length TBX18 was not able to recruit HAUS8, ASAP and PARP3 to the nucleus (Fig 6C). Finally, we wished to ascertain that TBX18 does not localize to the centrosome. For this, we transfected an expression construct for full-length MYC-tagged TBX18 in 293 cells and performed co-immunofluorescence analysis of the MYC tag and the centrosomal marker protein TUBG (gamma-Tubulin) [37]. TBX18 protein was not observed at the centrosome but exclusively localized to the nucleus (Fig 6D). We conclude that TBX18 is able to interact with some of the proteins annotated as “centrosomal” in 293 cells (B9D2, TPX2) but that this interaction occurs in the nucleus.

## Discussion

The tandem affinity purification (TAP)-Tag method has been successfully used to identify interactome members of different proteins in 293 cells [38, 39]. Here, we used this approach with dual tagged overexpressed TBX18 and identified protein interaction partners of TBX18 in 293 cells. Our results show that TBX18 preferentially binds to chromatin and interacts with transcriptional cofactors (preferentially corepressors) and homeobox transcription factors. Binding to proteins annotated as “centrosomal” may reflect an unknown nuclear function of these proteins rather than a novel centrosomal role of TBX18.

We have previously shown that TBX18 interacts with members of the TLE (Groucho) family via a conserved eh1-motif and that this interaction accounts for half of the repressive activity of TBX18 in transactivation assays *in vitro* [21]. TBX18 protein with loss of the C-terminal region and unchanged eh1-motif from a CAKUT patient exhibited reduced repressive activity *in vitro* [24] indicating that additional corepressors exist both in 293 cells as well as *in vivo* and that they may bind to the large C-terminal region of TBX18 to augment its repressive activity. Amongst 10 transcriptional cofactors identified in our proteomic screen, we validated robust and probably direct binding of 6 proteins (CBFB, CHD7, GAR1, IKZF2, NCOA5, SBNO2) to TBX18. Only CBFB interacted weakly with the C-terminal region, while all proteins bound to the T-box. This result was somehow disappointing to us since we had hoped to identify a C-terminally binding repressor that may explain the human disease mechanism. It is important to note that our proteomic approach did not reach saturation since we only detected a single protein in more than one of the three independent IP experiments. The list of cofactors and interactors, respectively, is therefore unlikely to be complete but may represent cofactors that bind relatively strongly to TBX18. Alternatively, the cofactor that operates in the embryonic mesenchyme of the ureter may simply be not present in 293 cells. Given the finding that both our candidate cofactors and the identified homeobox transcription factors interacted with the T-box, the argument may arise that the identified cofactors may bind indirectly e.g. via the homeobox factors to TBX18. While this may be well the case in cells, we assume that the interaction between GTS-TBX18 fusion proteins from bacteria and *in vitro* translated proteins from reticulocytes lacks such mediators, and therefore, is direct.

We identified three cofactors that bound to TBX18 and variably enhanced the repressive activity of TBX18: CBFB, CHD7 and IKZF2. CBFB represents the beta subunit of the core-binding factor protein. The alpha subunit is encoded by one of three RUNX proteins. The complex acts as a transcription factor with CBFB increasing the affinity of the alpha subunit for DNA and enhancing transcriptional activation by RUNX proteins [40]. Compatible with this function CBFB only poorly affected the transcriptional repression activity of TBX18.

CHD7 is a member of the Chromodomain Helicase DNA-binding (CHD) protein family that acts as ATP-dependent chromatin remodeler [41]. CHD7 was shown to bind to TBX20, a protein closely related to TBX18 [42], and to genetically interact with *TBX1*, another related family member in the development of the great vessels [43]. Interestingly, a mutation in amino acid residue 1684 of CHD7, was identified as a penetrant cause for CAKUT [44], and CHD7 was found to bind to CBFB through RUNX1 to repress RUNX1-induced genes [45]. Additionally, CHD4, another CHD family member that is a part of the Mi-2/NuRD (Nucleosome Remodeling Deacetylase) complex [46, 47] interacts with IKZF2 extensively during blood cell development [48–50]. IKZF2 is a member of the Ikaros Zn-finger containing DNA-binding protein family (thus not a classical cofactor) that additionally interacts with HDACs [51]. Together with the enhanced repressive activity of TBX18 by IKZF2, this suggests that TBX18 forms a complex with members of the CHD family (CHD4, CHD7 or others), the Ikaros family, CBFB and HDACs to compact chromatin and deacetylate histones for repression.

SBNO2 forms together with SBNO1 the small family of DExD/H helicase Strawberry notch-like proteins. SBNO2 can repress transcription of genes [52] but also activate [53] indicating a context-dependent role as transcriptional cofactor. Similarly, NCOA5, a factor that regulates nuclear receptor transcriptional activity, was shown to negatively and positively impact on gene expression [54, 55]. In our transcription assays both proteins relieved the transcriptional repression by TBX18 indicating that they act as transcriptional activators. This does not exclude a role as a repressor *in vivo*, given the fact that differentially spaced and oriented TBEs can translate into different transcriptional responses [4].

GAR1 is a component of box H/ACA ribonucleoproteins (RNPs), protein-RNA complexes responsible for post-transcriptional modification of cellular RNAs, splicing and ribosome synthesis [56] explaining the lack of this protein to affect transcription. The possible significance of TBX18 interaction remains unclear.

Previous work showed that *in vitro* TBX18 interacts with at least three different classes of tissue specific transcription factors, namely Zn-finger proteins (GATA4), T-box proteins (TBX15, TBX18) and homeobox proteins (NKX2.5, PAX3 and SIX1) [21–23]. We were surprised to identify in our proteomic screen in 293 only homeobox containing transcription factors. All of them interacted with the T-box of TBX18 in pull-down assays. Although this may indicate a general promiscuity of T-box interaction with this class of proteins, it seems likely that certain subfamilies of homeobox proteins are preferred interaction partners *in vivo* as well. Our screen identified as a binding partner, PAX1, a member of the paired-type homeobox transcription factors [57]. Interestingly, we have previously shown that another member of this family, PAX3 binds to TBX18, and that loss-of-function alleles of *Pax3* and *Tbx18* genetically interact in limb girdle development [22]. Although *Pax1* is not coexpressed with *Tbx18* in the ureteric mesenchyme, *Pax1*, *Pax9* or other family members may bind to *TBX18* in other embryological contexts including the somitic mesoderm in which they are partially coexpressed [17].

We also identified two of the four members of the PBX subfamily (PBX1, PBX4) as TBX18 binding partners *in vitro* [58]. Three members of this subfamily (*Pbx1*, *Pbx2*, *Pbx3*) are coexpressed with *Tbx18* in the ureteric mesenchyme. Importantly, a set of mutations recently identified *Pbx1* as monogenic cause of CAKUT making a functional interaction with TBX18 in ureter development likely [44]. Other candidates for functional interaction are the two *Prrx* genes that are coexpressed with *Tbx18* in the ureteric mesenchyme but also in the developing limb [59].

Since a recent report ascribed an additional centrosomal function to the transcription factor ATF [35], we were excited that functional annotation and literature mining identified a cluster of centrosomal proteins as binding candidates of TBX18 in our proteomic screen in 293 cells. We validated direct binding of 6 of these candidates to the T-box of TBX18, and confirmed interaction of two of them, B9D2 and TPX2, with TBX18 in 293 cells. To our surprise, B9D2, which was reported as a ciliary transition zone protein [60], was mainly located in the cytoplasm of 293 cells, while TPX2 a microtubule binding protein that is necessary for spindle assembly was found in the nucleus. Interestingly, it was recently reported that TPX2 resides preferentially in the nucleus during interphase where it plays a role in amplification of the DNA damage response [61, 62]. Since TBX18 does not localize to the centrosome even when overexpressed in 293 cells, we deem a centrosomal function of TBX18 unlikely, but suggest that ciliary or microtubule-binding proteins may be shuttled to the nucleus to affect the function of transcription factors such as TBX18.

Together, our proteomic screen strongly supports an exclusive nuclear function of TBX18 as a repressor of chromatin accessibility. At least in 293 cells, this function is likely mediated by recruiting chromatin remodeling complexes of the CHD family and histone modifying complexes including HDACs in concert with other transcription factors, most likely of the homeobox family. Whether these complexes are conserved at the different sites of TBX18 expression in the embryo, will be a demanding yet exciting task to find out for genetic interaction screens and proteomic approaches with endogenously tagged TBX18 in the future.

## Supporting information

S1 Fig *Tbx18* is expressed in 293 cells but not in other tested cell lines. Semi-quantitative RT-PCR analysis of *TBX18* expression in 293, SK-N-MC, SW-13, Huh-7.5, SH-SY5Y and 786-O cells. *GAPDH* was used as a housekeeping control and all values were calculated as relative gene per *GAPDH* ratios. 293 cells exhibit a strong expression of *TBX18* with a mean ± SD ratio of 1.1408±0.2394. In all other tested cell lines *TBX18* expression was not above background level.
(TIF)

S2 Fig Expression and localization of identified transcriptional cofactor candidates in 293 cells. (A) Western Blot analysis of over-expressed co-factors in 293 cells. Expression constructs (as listed in S5A Table) were transfected into 293 cells and exogenous proteins were detected using antibodies against the corresponding tags. All detected proteins were of the expected size except BASP1, which as previously appeared larger. (B) Immunofluorescence analysis of localization of candidate transcriptional cofactors after transfection of 293 cells with the expression plasmids listed in (A). BASP1, CHD7, GAR1, IKZF2, NCOA5, SBNO2, SSXB2, SUV39H2 localized to the nucleus, CBFB and RCOR3 to the cytoplasm. Scale bar length is 25 μm.
(TIF)

S3 Fig GST-TBX18 fusion proteins for pull-down assays. (A) Schematic representation of the primary structure of TBX18, and of the subfragments used to express GST fusion proteins. The T-box (T) is shaded in orange, and the N- and C-terminal domains (N and C) are shown in grey. The numbers refer to the amino acid position in the full-length TBX18 protein. The localization of the Groucho binding region (eh1), the nuclear localization signal (NLS) are highlighted in the N-terminal domain. (B) GST and fusion proteins of GST and N-, N+T-, T- and C-domains of TBX18 were purified from *E*. *coli* extracts and analyzed for integrity and quantity by Coomassie Brilliant Blue staining of SDS-polyacrylamide gels. Asterisks mark the full-length proteins.
(TIF)

S4 Fig Western Blot analysis of over-expressed “centrosomal” candidate proteins in 293 cells. Expression constructs (as listed in S4E Table) were transfected into 293 cells and exogenous proteins were detected using antibodies against the corresponding tags.
(TIF)

S1 Table Summary of cloning strategies for expression plasmids. (XLSX)

S2 Table Mass spectrometry analysis identifies proteins copurified with TBX18 from 293 cells. List of 143 proteins detected by mass spectrometry in extracts of proteins copurified with TBX18 in 293 cells. Shown are Uniprot accession code (first row), the protein symbol (second row) and the protein name (third row). Color code identifies the three major protein clusters identified: transcription factors of the homeobox family (grey), transcriptional cofactors (green) and centrosomal proteins (yellow).
(XLSX)

S3 Table DAVID analysis identifies 83 enriched functional annotations in the set of 143 proteins identified as possible TBX18 interaction partners in 293 cells. From the 143 identified proteins we excluded immunoglobulins, histone proteins and cytoskeletal proteins which leads to 83 possible interaction partners.
(XLSX)

S4 Table DAVID analysis identifies 5 enriched clusters of TBX18 interacting proteins. All 143 identified proteins were subjected to a Functional Annotation Clustering (DAVID Bioinformatics Resources 6.8) using the following settings: Similarity Term Overlap 7; Similarity Threshold 0.35; Initial Group Membership 2; Final Group Membership 2; Multiple Linkage Threshold 0.15; EASE 0.3; UP_KEYWORDS and GOTHERM_CC_DIRECT. 5 clusters were identified with enrichment scores for cluster 1 of 6.07, for cluster 2 of 3.48, for cluster 3 of 1.85, for cluster 4 of 1.35 and cluster 5 of 1.19.
(XLSX)

S5 Table Summary of expression of candidate proteins for TBX18 interaction in various assays. (A) Cloning and expression of candidate transcriptional cofactors in 293 cells. Shown are the name of the proteins, the species they are derived from (m, mouse; h, human), the expected molecular weight (in kDa), the plasmids used for expression of the proteins in 293 cells, the antibodies used to detect the tag on the candidate proteins, and the reference for the used plasmids. (B-D) Cloning and *in vitro* translation of candidate transcriptional cofactors (B), homeobox transcription factors (C) and centrosomal proteins (D). Shown are the name of the proteins, the species they are derived from (m, mouse; h, human), the expected molecular weight (in kDa), the plasmids used for expression of the proteins in reticulocyte lysate, the polymerase used for the *in vitro* transcription reaction, and the reference for the used plasmids. (E) Cloning and expression of candidate “centrosomal” proteins in 293 cells. Shown are the name of the proteins, the species they are derived from (m, mouse; h, human), the expected molecular weight (in kDa), the plasmids used for expression of the proteins in 293 cells, the antibodies used to detect the tag on the candidate proteins, and the reference for the used plasmids.
(XLSX)

S6 Table Statistical analysis of transactivation assays of candidate transcriptional cofactors with TBX18 in 293 cells. Shown are the experimental setup for each sample of the transactivation assays and the statistical analysis performed to the transactivation assay results. Raw luminescence data was normalized to the average of the reporter alone measurements. Further, average and standard deviation were quantified from the two technical and two experimental replicates. The significance of luciferase expression between the TBX18 induced repression and the reporter and the effects of the cofactors on TBX18 repression was evaluated by a student t-test (TTEST) carried out according to an analysis of variance equality (FTEST). p-values<0.05 (*) were considered significant, while p-values<0.01 (**) and p-values<0.0001 (***) were considered very significant.
(XLSX)

## References

[1] Bollag RJ, Siegfried Z, Cebra-Thomas JA, Garvey N, Davison EM, Silver LM. An ancient family of embryonically expressed mouse genes sharing a conserved protein motif with the T locus. Nat Genet. 1994;7: 383–9. 10.1038/ng0794-383 7920656

[2] Agulnik SI, Bollag RJ, Silver LM. Conservation of the T-box gene family from Mus musculus to Caenorhabditis elegans. Genomics. 1995;25: 214–9. 7774921 10.1016/0888-7543(95)80128-9

[3] Kispert A, Herrmann BG. The Brachyury gene encodes a novel DNA binding protein. EMBO J. 1993;12: 3211–20. 8344258 10.1002/j.1460-2075.1993.tb05990.x PMC413588

[4] Kispert A, Koschorz B, Herrmann BG. The T protein encoded by Brachyury is a tissue-specific transcription factor. EMBO J. 1995;14: 4763–72. 7588606 10.1002/j.1460-2075.1995.tb00158.x PMC394574

[5] Naiche LA, Harrelson Z, Kelly RG, Papaioannou VE. T-box genes in vertebrate development. Annu Rev Genet. 2005;39: 219–39. 10.1146/annurev.genet.39.073003.105925 16285859

[6] Papaioannou VE. The T-box gene family: emerging roles in development, stem cells and cancer. Development. 2014;141: 3819–33. 10.1242/dev.104471 25294936 PMC4197708

[7] Ghosh TK, Brook JD, Wilsdon A. T-Box Genes in Human Development and Disease. Curr Top Dev Biol. 2017;122: 383–415. 10.1016/bs.ctdb.2016.08.006 28057271

[8] Sebe-Pedros A, Ruiz-Trillo I. Evolution and Classification of the T-Box Transcription Factor Family. Curr Top Dev Biol. 2017;122: 1–26. 10.1016/bs.ctdb.2016.06.004 28057261

[9] Kraus F, Haenig B, Kispert A. Cloning and expression analysis of the mouse T-box gene Tbx18. Mech Dev. 2001;100: 83–6. 11118889 10.1016/s0925-4773(00)00494-9

[10] Bush JO, Lan Y, Maltby KM, Jiang R. Isolation and developmental expression analysis of Tbx22, the mouse homolog of the human X-linked cleft palate gene. Dev Dyn. 2002;225: 322–6. 10.1002/dvdy.10154 12412015

[11] Herr A, Meunier D, Muller I, Rump A, Fundele R, Ropers HH, et al Expression of mouse Tbx22 supports its role in palatogenesis and glossogenesis. Dev Dyn. 2003;226: 579–86. 10.1002/dvdy.10260 12666195

[12] Singh MK, Petry M, Haenig B, Lescher B, Leitges M, Kispert A. The T-box transcription factor Tbx15 is required for skeletal development. Mech Dev. 2005;122: 131–44. 10.1016/j.mod.2004.10.011 15652702

[13] Airik R, Bussen M, Singh MK, Petry M, Kispert A. Tbx18 regulates the development of the ureteral mesenchyme. J Clin Invest. 2006;116: 663–74. 10.1172/JCI26027 16511601 PMC1386107

[14] Christoffels VM, Mommersteeg MT, Trowe MO, Prall OW, de Gier-de Vries C, Soufan AT, et al Formation of the venous pole of the heart from an Nkx2-5-negative precursor population requires Tbx18. Circ Res. 2006;98: 1555–63. 10.1161/01.RES.0000227571.84189.65 16709898

[15] Trowe MO, Maier H, Schweizer M, Kispert A. Deafness in mice lacking the T-box transcription factor Tbx18 in otic fibrocytes. Development. 2008;135: 1725–34. 10.1242/dev.014043 18353863

[16] Bolt CC, Negi S, Guimaraes-Camboa N, Zhang H, Troy JM, Lu X, et al Tbx18 Regulates the Differentiation of Periductal Smooth Muscle Stroma and the Maintenance of Epithelial Integrity in the Prostate. PLoS One. 2016;11: e0154413 10.1371/journal.pone.0154413 27120339 PMC4847854

[17] Bussen M, Petry M, Schuster-Gossler K, Leitges M, Gossler A, Kispert A. The T-box transcription factor Tbx18 maintains the separation of anterior and posterior somite compartments. Genes Dev. 2004;18: 1209–21. 10.1101/gad.300104 15155583 PMC415645

[18] Wiese C, Grieskamp T, Airik R, Mommersteeg MT, Gardiwal A, de Gier-de Vries C, et al Formation of the sinus node head and differentiation of sinus node myocardium are independently regulated by Tbx18 and Tbx3. Circ Res. 2009;104: 388–97. 10.1161/CIRCRESAHA.108.187062 19096026

[19] Greulich F, Farin HF, Schuster-Gossler K, Kispert A. Tbx18 function in epicardial development. Cardiovasc Res. 2012;96: 476–83. 10.1093/cvr/cvs277 22926762

[20] Wu SP, Dong XR, Regan JN, Su C, Majesky MW. Tbx18 regulates development of the epicardium and coronary vessels. Dev Biol. 2013;383: 307–20. 10.1016/j.ydbio.2013.08.019 24016759 PMC4172450

[21] Farin HF, Bussen M, Schmidt MK, Singh MK, Schuster-Gossler K, Kispert A. Transcriptional repression by the T-box proteins Tbx18 and Tbx15 depends on Groucho corepressors. J Biol Chem. 2007;282: 25748–59. 10.1074/jbc.M703724200 17584735

[22] Farin HF, Mansouri A, Petry M, Kispert A. T-box protein Tbx18 interacts with the paired box protein Pax3 in the development of the paraxial mesoderm. J Biol Chem. 2008;283: 25372–80. 10.1074/jbc.M802723200 18644785

[23] Nie X, Sun J, Gordon RE, Cai CL, Xu PX. SIX1 acts synergistically with TBX18 in mediating ureteral smooth muscle formation. Development. 2010;137: 755–65. 10.1242/dev.045757 20110314 PMC2827686

[24] Vivante A, Kleppa MJ, Schulz J, Kohl S, Sharma A, Chen J, et al Mutations in TBX18 Cause Dominant Urinary Tract Malformations via Transcriptional Dysregulation of Ureter Development. Am J Hum Genet. 2015;97: 291–301. 10.1016/j.ajhg.2015.07.001 26235987 PMC4862256

[25] Tanimizu N, Tsujimura T, Takahide K, Kodama T, Nakamura K, Miyajima A. Expression of Dlk/Pref-1 defines a subpopulation in the oval cell compartment of rat liver. Gene Expr Patterns. 2004;5: 209–18. 10.1016/j.modgep.2004.08.003 15567716

[26] Schneider CA, Rasband WS, Eliceiri KW. NIH Image to ImageJ: 25 years of image analysis. Nat Methods. 2012;9: 671–5. 22930834 10.1038/nmeth.2089 PMC5554542

[27] Zirzow S, Ludtke TH, Brons JF, Petry M, Christoffels VM, Kispert A. Expression and requirement of T-box transcription factors Tbx2 and Tbx3 during secondary palate development in the mouse. Dev Biol. 2009;336: 145–55. 10.1016/j.ydbio.2009.09.020 19769959

[28] O'Brien LL, Guo Q, Bahrami-Samani E, Park JS, Hasso SM, Lee YJ, et al Transcriptional regulatory control of mammalian nephron progenitors revealed by multi-factor cistromic analysis and genetic studies. PLoS Genet. 2018;14: e1007181 10.1371/journal.pgen.1007181 29377931 PMC5805373

[29] Pear WS, Nolan GP, Scott ML, Baltimore D. Production of high-titer helper-free retroviruses by transient transfection. Proc Natl Acad Sci U S A. 1993;90: 8392–6. 7690960 10.1073/pnas.90.18.8392 PMC47362

[30] Wang J, Cantor AB, Orkin SH. Tandem affinity purification of protein complexes in mouse embryonic stem cells using in vivo biotinylation. Curr Protoc Stem Cell Biol. 2009;Chapter 1: Unit1B 5. 10.1002/9780470151808.sc01b05s8 PMC2782545 19306258

[31] Jochim N, Gerhard R, Just I, Pich A. Impact of clostridial glucosylating toxins on the proteome of colonic cells determined by isotope-coded protein labeling and LC-MALDI. Proteome Sci. 2011;9: 48 10.1186/1477-5956-9-48 21849038 PMC3176154

[32] Cox J, Mann M. MaxQuant enables high peptide identification rates, individualized p.p.b.-range mass accuracies and proteome-wide protein quantification. Nat Biotechnol. 2008;26: 1367–72. 10.1038/nbt.1511 19029910

[33] Huang da W, Sherman BT, Lempicki RA. Systematic and integrative analysis of large gene lists using DAVID bioinformatics resources. Nat Protoc. 2009;4: 44–57. 10.1038/nprot.2008.211 19131956

[34] Moorman AF, Houweling AC, de Boer PA, Christoffels VM. Sensitive nonradioactive detection of mRNA in tissue sections: novel application of the whole-mount in situ hybridization protocol. J Histochem Cytochem. 2001;49: 1–8. 10.1177/002215540104900101 11118473

[35] Madarampalli B, Yuan Y, Liu D, Lengel K, Xu Y, Li G, et al ATF5 Connects the Pericentriolar Materials to the Proximal End of the Mother Centriole. Cell. 2015;162: 580–92. 10.1016/j.cell.2015.06.055 26213385

[36] Schulz Y, Freese L, Manz J, Zoll B, Volter C, Brockmann K, et al CHARGE and Kabuki syndromes: a phenotypic and molecular link. Hum Mol Genet. 2014;23: 4396–405. 10.1093/hmg/ddu156 24705355

[37] Graf R, Euteneuer U, Ueda M, Schliwa M. Isolation of nucleation-competent centrosomes from Dictyostelium discoideum. Eur J Cell Biol. 1998;76: 167–75. 10.1016/S0171-9335(98)80031-9 9716263

[38] Gloeckner CJ, Kinkl N, Schumacher A, Braun RJ, O'Neill E, Meitinger T, et al The Parkinson disease causing LRRK2 mutation I2020T is associated with increased kinase activity. Hum Mol Genet. 2006;15: 223–32. 10.1093/hmg/ddi439 16321986

[39] den Hollander AI, Koenekoop RK, Mohamed MD, Arts HH, Boldt K, Towns KV, et al Mutations in LCA5, encoding the ciliary protein lebercilin, cause Leber congenital amaurosis. Nat Genet. 2007;39: 889–95. 10.1038/ng2066 17546029

[40] Yoshida CA, Furuichi T, Fujita T, Fukuyama R, Kanatani N, Kobayashi S, et al Core-binding factor beta interacts with Runx2 and is required for skeletal development. Nat Genet. 2002;32: 633–8. 10.1038/ng1015 12434152

[41] Bouazoune K, Kingston RE. Chromatin remodeling by the CHD7 protein is impaired by mutations that cause human developmental disorders. Proc Natl Acad Sci U S A. 2012;109: 19238–43. 10.1073/pnas.1213825109 23134727 PMC3511097

[42] Kennedy L, Kaltenbrun E, Greco TM, Temple B, Herring LE, Cristea IM, et al Formation of a TBX20-CASZ1 protein complex is protective against dilated cardiomyopathy and critical for cardiac homeostasis. PLoS Genet. 2017;13: e1007011 10.1371/journal.pgen.1007011 28945738 PMC5629033

[43] Randall V, McCue K, Roberts C, Kyriakopoulou V, Beddow S, Barrett AN, et al Great vessel development requires biallelic expression of Chd7 and Tbx1 in pharyngeal ectoderm in mice. J Clin Invest. 2009;119: 3301–10. 10.1172/JCI37561 19855134 PMC2769172

[44] Heidet L, Moriniere V, Henry C, De Tomasi L, Reilly ML, Humbert C, et al Targeted Exome Sequencing Identifies PBX1 as Involved in Monogenic Congenital Anomalies of the Kidney and Urinary Tract. J Am Soc Nephrol. 2017;28: 2901–14. 10.1681/ASN.2017010043 28566479 PMC5619971

[45] Zhen T, Kwon EM, Zhao L, Hsu J, Hyde RK, Lu Y, et al Chd7 deficiency delays leukemogenesis in mice induced by Cbfb-MYH11. Blood. 2017;130: 2431–42. 10.1182/blood-2017-04-780106 29018080 PMC5709785

[46] Low JK, Webb SR, Silva AP, Saathoff H, Ryan DP, Torrado M, et al CHD4 Is a Peripheral Component of the Nucleosome Remodeling and Deacetylase Complex. J Biol Chem. 2016;291: 15853–66. 10.1074/jbc.M115.707018 27235397 PMC4957066

[47] Kolla V, Naraparaju K, Zhuang T, Higashi M, Kolla S, Blobel GA, et al The tumour suppressor CHD5 forms a NuRD-type chromatin remodelling complex. Biochem J. 2015;468: 345–52. 10.1042/BJ20150030 25825869 PMC4487910

[48] Bottardi S, Mavoungou L, Pak H, Daou S, Bourgoin V, Lakehal YA, et al The IKAROS interaction with a complex including chromatin remodeling and transcription elongation activities is required for hematopoiesis. PLoS Genet. 2014;10: e1004827 10.1371/journal.pgen.1004827 25474253 PMC4256266

[49] O'Neill DW, Schoetz SS, Lopez RA, Castle M, Rabinowitz L, Shor E, et al An ikaros-containing chromatin-remodeling complex in adult-type erythroid cells. Mol Cell Biol. 2000;20: 7572–82. 11003653 10.1128/mcb.20.20.7572-7582.2000 PMC86310

[50] Kim J, Sif S, Jones B, Jackson A, Koipally J, Heller E, et al Ikaros DNA-binding proteins direct formation of chromatin remodeling complexes in lymphocytes. Immunity. 1999;10: 345–55. 10204490 10.1016/s1074-7613(00)80034-5

[51] Fan Y, Lu D. The Ikaros family of zinc-finger proteins. Acta Pharm Sin B. 2016;6: 513–21. 10.1016/j.apsb.2016.06.002 27818917 PMC5071621

[52] El Kasmi KC, Smith AM, Williams L, Neale G, Panopoulos AD, Watowich SS, et al Cutting edge: A transcriptional repressor and corepressor induced by the STAT3-regulated anti-inflammatory signaling pathway. J Immunol. 2007;179: 7215–9. 18025162 10.4049/jimmunol.179.11.7215

[53] Maruyama K, Uematsu S, Kondo T, Takeuchi O, Martino MM, Kawasaki T, et al Strawberry notch homologue 2 regulates osteoclast fusion by enhancing the expression of DC-STAMP. J Exp Med. 2013;210: 1947–60. 10.1084/jem.20130512 23980096 PMC3782043

[54] Sauve F, McBroom LD, Gallant J, Moraitis AN, Labrie F, Giguere V. CIA, a novel estrogen receptor coactivator with a bifunctional nuclear receptor interacting determinant. Mol Cell Biol. 2001;21: 343–53. 10.1128/MCB.21.1.343-353.2001 11113208 PMC88807

[55] Gillespie MA, Gold ES, Ramsey SA, Podolsky I, Aderem A, Ranish JA. An LXR-NCOA5 gene regulatory complex directs inflammatory crosstalk-dependent repression of macrophage cholesterol efflux. EMBO J. 2015;34: 1244–58. 10.15252/embj.201489819 25755249 PMC4426483

[56] Leulliot N, Godin KS, Hoareau-Aveilla C, Quevillon-Cheruel S, Varani G, Henry Y, et al The box H/ACA RNP assembly factor Naf1p contains a domain homologous to Gar1p mediating its interaction with Cbf5p. J Mol Biol. 2007;371: 1338–53. 10.1016/j.jmb.2007.06.031 17612558

[57] Blake JA, Ziman MR. Pax genes: regulators of lineage specification and progenitor cell maintenance. Development. 2014;141: 737–51. 10.1242/dev.091785 24496612

[58] Capellini TD, Zappavigna V, Selleri L. Pbx homeodomain proteins: TALEnted regulators of limb patterning and outgrowth. Dev Dyn. 2011;240: 1063–86. 10.1002/dvdy.22605 21416555 PMC3081394

[59] Lu MF, Cheng HT, Lacy AR, Kern MJ, Argao EA, Potter SS, et al Paired-related homeobox genes cooperate in handplate and hindlimb zeugopod morphogenesis. Dev Biol. 1999;205: 145–57. 10.1006/dbio.1998.9116 9882503

[60] Zhao C, Malicki J. Nephrocystins and MKS proteins interact with IFT particle and facilitate transport of selected ciliary cargos. EMBO J. 2011;30: 2532–44. 10.1038/emboj.2011.165 21602787 PMC3155299

[61] Neumayer G, Belzil C, Gruss OJ, Nguyen MD. TPX2: of spindle assembly, DNA damage response, and cancer. Cell Mol Life Sci. 2014;71: 3027–47. 10.1007/s00018-014-1582-7 24556998 PMC11114040

[62] Neumayer G, Nguyen MD. TPX2 impacts acetylation of histone H4 at lysine 16: implications for DNA damage response. PLoS One. 2014;9: e110994 10.1371/journal.pone.0110994 25365214 PMC4217740

