# Supplementary figures and images for: Proteomic analysis identifies transcriptional cofactors and homeobox transcription factors as TBX18 binding proteins
Source: PLoS One. 2018 Aug 2;13(8):e0200964. doi: 10.1371/journal.pone.0200964 (PMC6071992; doi:10.1371/journal.pone.0200964)

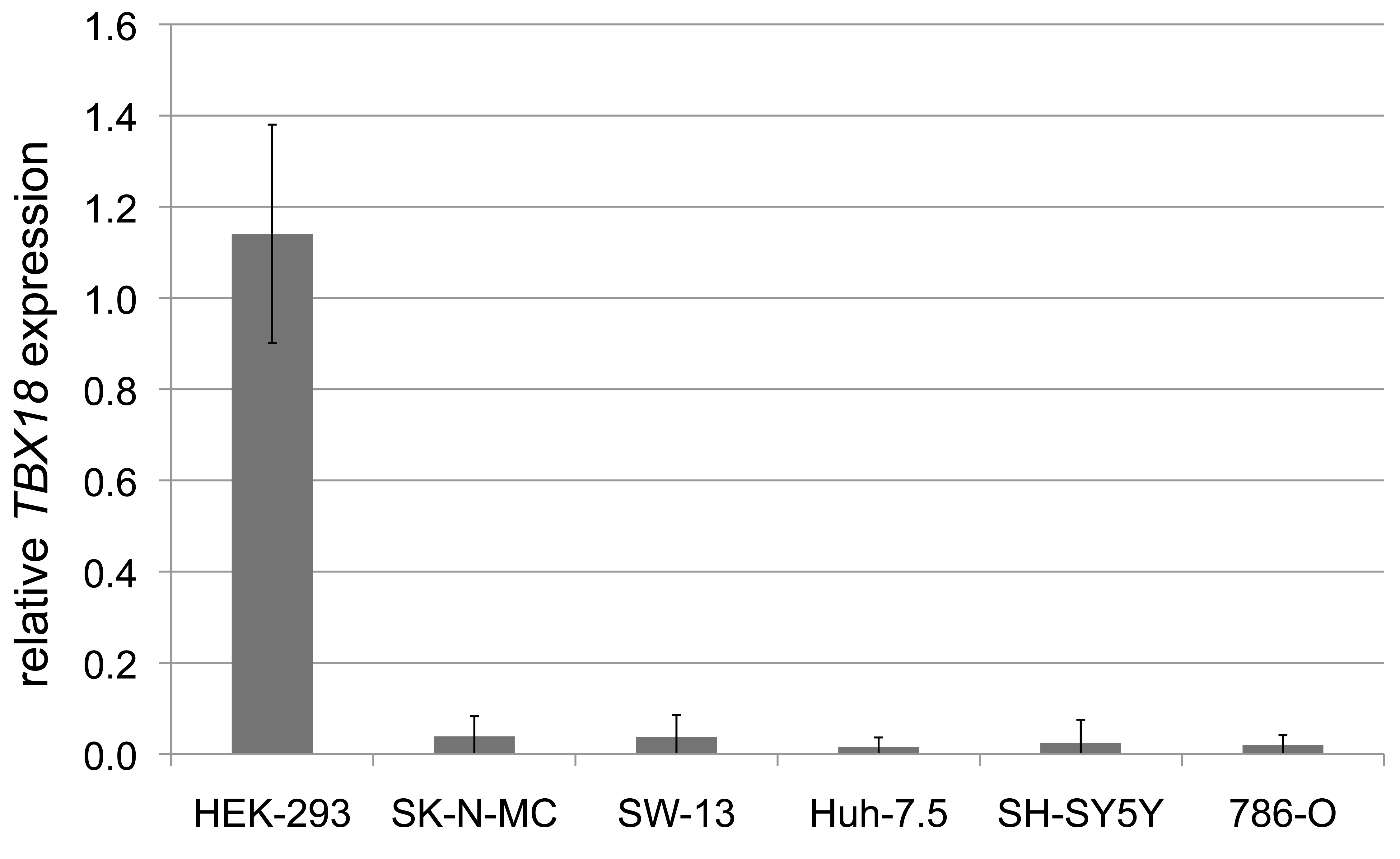

Supplement: S1 Fig — Semi-quantitative RT-PCR analysis of TBX18 expression in 293, SK-N-MC, SW-13, Huh-7.5, SH-SY5Y and 786-O cells. GAPDH was used as a housekeeping control and all values were calculated as relative gene per GAPDH ratios. 293 cells exhibit a strong expression of TBX18 with a mean ± SD ratio of 1.1408±0.2394. In all other tested cell lines TBX18 expression was not above background level. (TIF) [file pone.0200964.s001.tif]

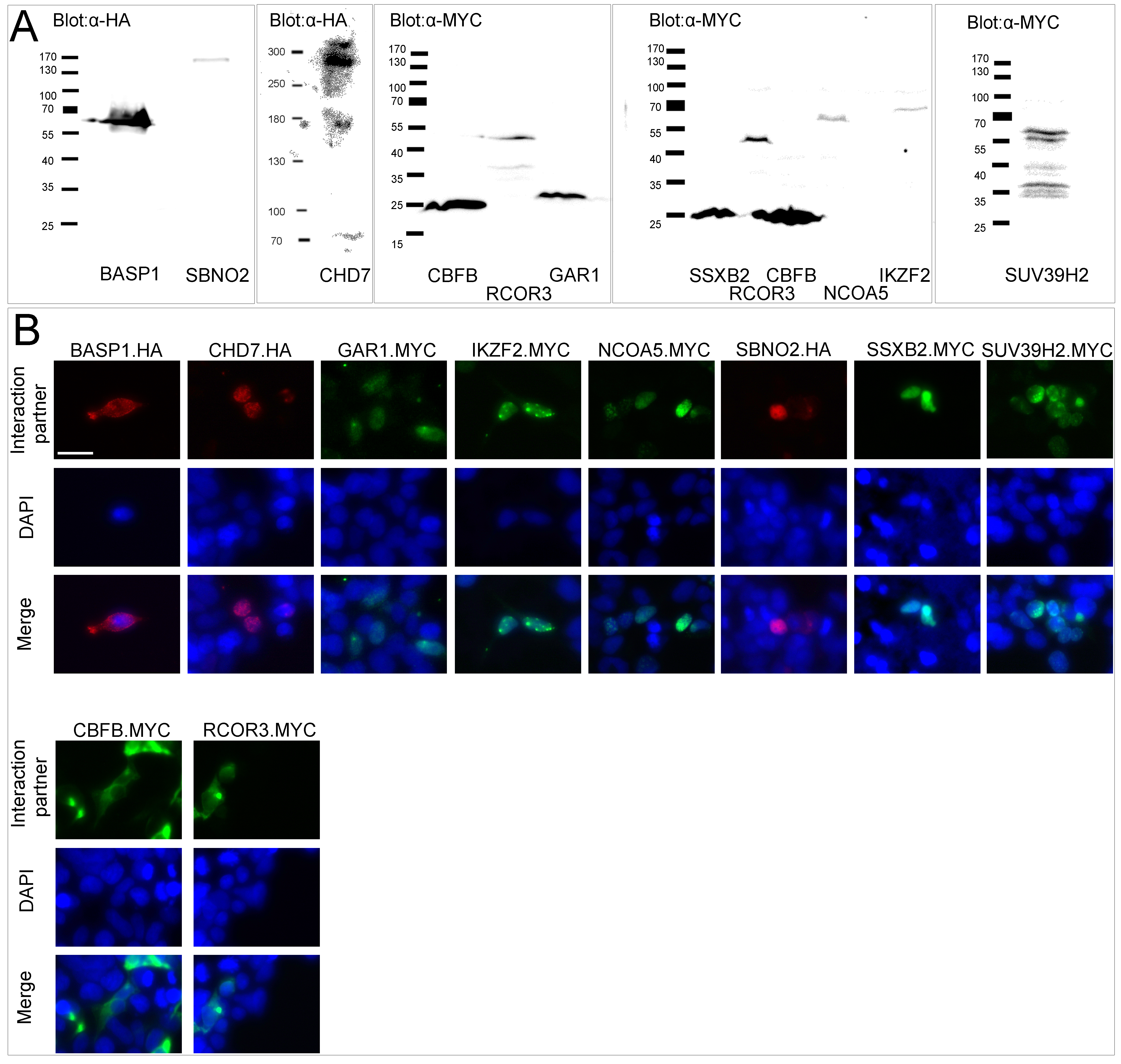

Supplement: S2 Fig — (A) Western Blot analysis of over-expressed co-factors in 293 cells. Expression constructs (as listed in S5A Table) were transfected into 293 cells and exogenous proteins were detected using antibodies against the corresponding tags. All detected proteins were of the expected size except BASP1, which as previously appeared larger. (B) Immunofluorescence analysis of localization of candidate transcriptional cofactors after transfection of 293 cells with the expression plasmids listed in (A). BASP1, CHD7, GAR1, IKZF2, NCOA5, SBNO2, SSXB2, SUV39H2 localized to the nucleus, CBFB and RCOR3 to the cytoplasm. Scale bar length is 25 μm. (TIF) [file pone.0200964.s002.tif]

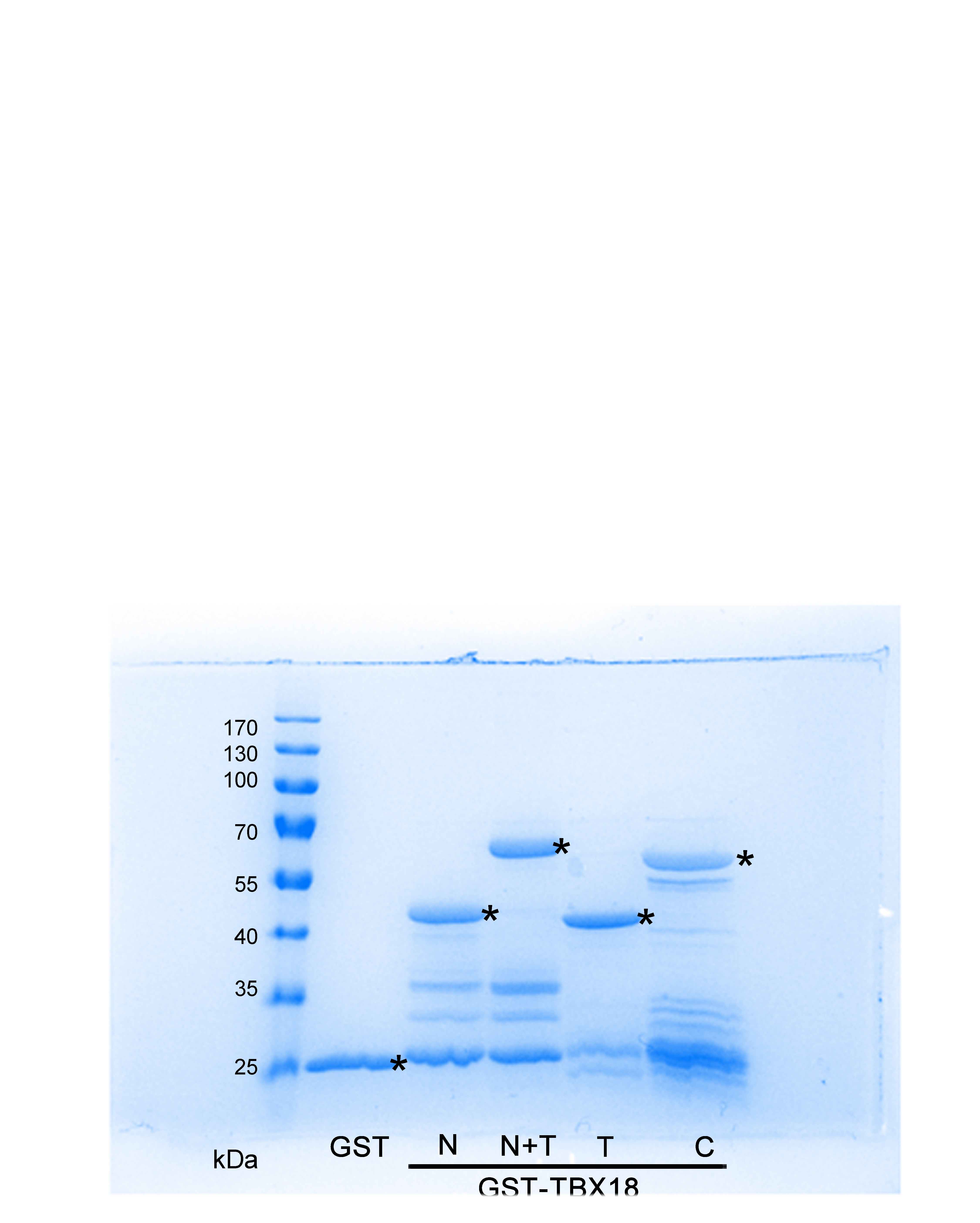

Supplement: S3 Fig — (A) Schematic representation of the primary structure of TBX18, and of the subfragments used to express GST fusion proteins. The T-box (T) is shaded in orange, and the N- and C-terminal domains (N and C) are shown in grey. The numbers refer to the amino acid position in the full-length TBX18 protein. The localization of the Groucho binding region (eh1), the nuclear localization signal (NLS) are highlighted in the N-terminal domain. (B) GST and fusion proteins of GST and N-, N+T-, T- and C-domains of TBX18 were purified from E. coli extracts and analyzed for integrity and quantity by Coomassie Brilliant Blue staining of SDS-polyacrylamide gels. Asterisks mark the full-length proteins. (TIF) [file pone.0200964.s003.tif]

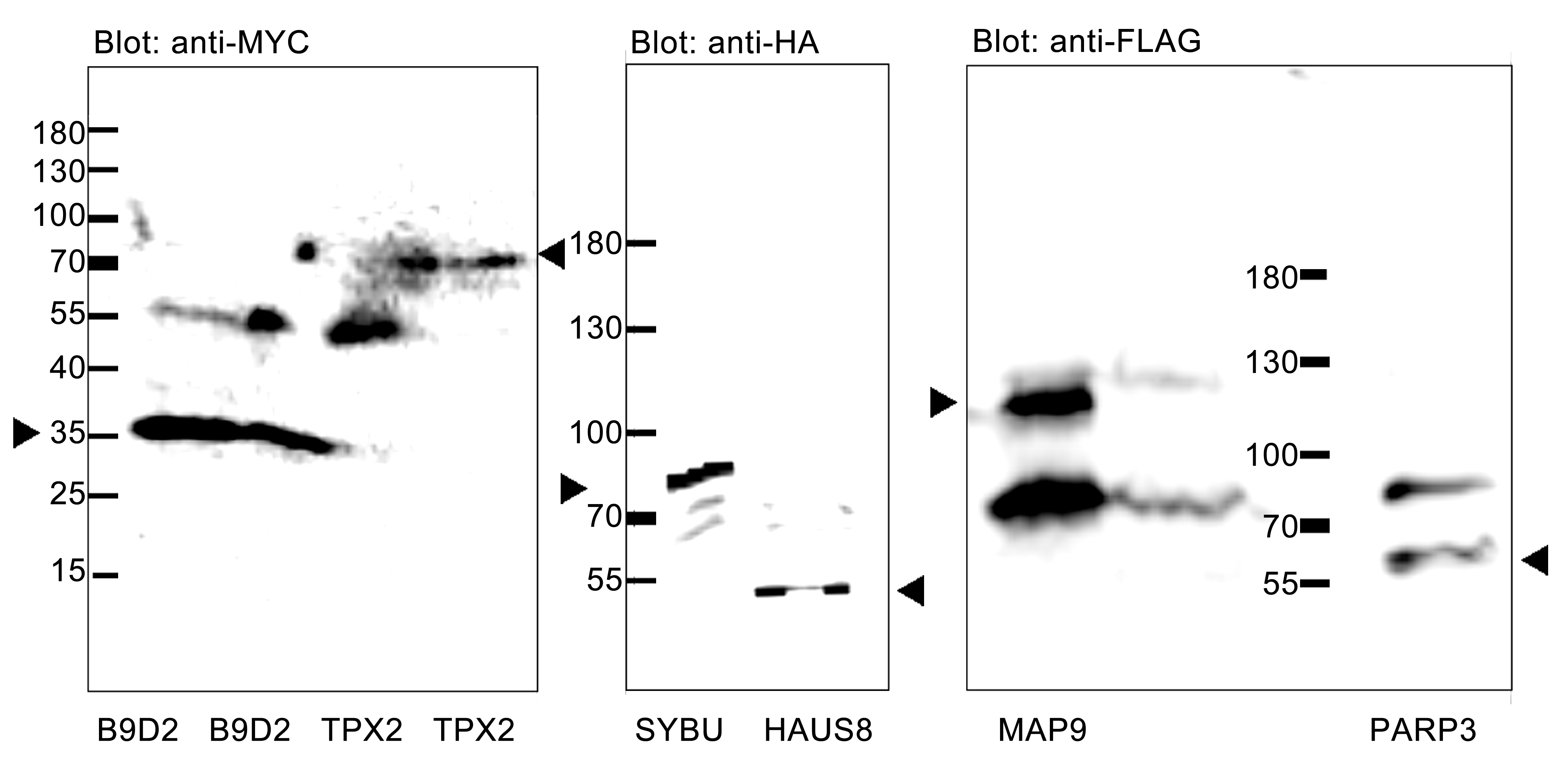

Supplement: S4 Fig — Expression constructs (as listed in S4E Table) were transfected into 293 cells and exogenous proteins were detected using antibodies against the corresponding tags. (TIF) [file pone.0200964.s004.tif]
